# Vacancy-defect modulated pathway of photoreduction of CO_2_ on single atomically thin AgInP_2_S_6_ sheets into olefiant gas

**DOI:** 10.1038/s41467-021-25068-7

**Published:** 2021-08-06

**Authors:** Wa Gao, Shi Li, Huichao He, Xiaoning Li, Zhenxiang Cheng, Yong Yang, Jinlan Wang, Qing Shen, Xiaoyong Wang, Yujie Xiong, Yong Zhou, Zhigang Zou

**Affiliations:** 1 Key Laboratory of Modern Acoustics (MOE), Institute of Acoustics, School of Physics, Jiangsu Key Laboratory of Nanotechnology, Eco-materials and Renewable Energy Research Center (ERERC), National Laboratory of Solid State Microstructures, Collaborative Innovation Center of Advanced Microstructures, Nanjing University, Nanjing, China; 2 School of Physics, Southeast University, Nanjing, China; 3 State Key Laboratory of Environmental Friendly Energy Materials, Southwest University of Science and Technology, Mianyang, China; 4 Institute of Superconducting & Electronic Materials, Innovation Campus, University of Wollongong, Squires Way, North Wollongong, NSW Australia; 5 Key Laboratory of Soft Chemistry and Functional Materials (MOE), Nanjing University of Science and Technology, Nanjing, China; 6 University of Electrocommunication, Grad Sch Informatics and Engineering, Chofu, Tokyo Japan; 7 Hefei National Laboratory for Physical Sciences at the Microscale, Collaborative Innovation Center of Chemistry for Energy Materials (iChEM), School of Chemistry and Materials Science, University of Science and Technology of China, Hefei, Anhui China; 8 School of Science and Engineering, The Chinese University of Hongkong (Shenzhen), Shenzhen, Guangdong China

**Keywords:** Photocatalysis, Carbon capture and storage, Solar fuels

## Abstract

Artificial photosynthesis, light-driving CO_2_ conversion into hydrocarbon fuels, is a promising strategy to synchronously overcome global warming and energy-supply issues. The quaternary AgInP_2_S_6_ atomic layer with the thickness of ~ 0.70 nm were successfully synthesized through facile ultrasonic exfoliation of the corresponding bulk crystal. The sulfur defect engineering on this atomic layer through a H_2_O_2_ etching treatment can excitingly change the CO_2_ photoreduction reaction pathway to steer dominant generation of ethene with the yield-based selectivity reaching ~73% and the electron-based selectivity as high as ~89%. Both DFT calculation and *in-situ* FTIR spectra demonstrate that as the introduction of S vacancies in AgInP_2_S_6_ causes the charge accumulation on the Ag atoms near the S vacancies, the exposed Ag sites can thus effectively capture the forming *CO molecules. It makes the catalyst surface enrich with key reaction intermediates to lower the C-C binding coupling barrier, which facilitates the production of ethene.

## Abstract (web summary)

CO_2_ conversion driven by light is a promising strategy to synchronously overcome global warming and energy-supply issues. Here the authors show that the sulfur defect engineering on a quaternary AgInP2S6 atomic layer can excitingly change the CO_2_ photoreduction reaction pathway to the generation of ethene.

## Introduction

Photocatalytic conversion of CO_2_ with H_2_O into solar fuels would be like killing two birds with one stone in terms of saving supplying energy and environment, which occurs mostly on the surfaces of semiconductors through complicated processes involving multi-electrons/protons transfer reactions^1^. Photo-driving CO_2_ hydrogenation into C_1_ species have been well achieved in the recent decade^2^, and our group has exploited a series of promising photocatalysts to converse CO_2_ to selectively form specific hydrocarbons, such as Zn_2_GeO_4_ ultrathin nanoribbons for CH_4_^3^, atomically thin InVO_4_ nanosheets for CO^4^, and TiO_2_-graphene hybrid nanosheets for C_2_H_6_^5^ and so on. However, the controlled C–C coupling to produce high-value C_2_ or C_2+_ products still remains a great challenge. Olefiant gas (ethylene, C_2_H_4_) is a chemical source of particular importance due to its high demand in the chemical industry. C_2_H_4_ is usually derived from steam cracking of naphtha under harsh production conditions (800–900 °C). It is definitely desirable for the realization of C_2_H_4_ synthesis through mild and environmentally benign pathways^6^.

Transition metal thio/selenophosphates (TPS) is a broad class of van der Waals layered structures with two sulfur or selenium layers sandwiching a layer of metal ions and P_2_ pairs and general compositions of M_4_[P_2_X_6_]^4−^, [M^2+^]_2_[P_2_X_6_]^4−^, and M^1+^M^3+^[P_2_X_6_]^4−^, where M^1+^ = Cu, Ag; M^3+^ = Cr, V, Al, Ga, etc. X = S, Se^7^. Those quaternary compounds exhibit mixed electron–ionic conductivity, promising optical and thermoelectric properties^8^. AgInP_2_S_6_ is a typical TPS with a rhombohedral structure and contains a sulfur framework with the octahedral voids filled by Ag, In, and P–P triangular patterns. Each AgInP_2_S_6_ monolayer consists of the [P_2_S_6_] anionic complex and two metallic cations (Ag and In) located at the center of sulfur near-octahedral polyhedrons connected one with the other by edges. Semiconducting AgInP_2_S_6_ crystal possesses an appropriate bandgap structure (*E*_g_ = ~2.4 eV), which is favored for visible light absorption^9^. The low value of the effective mass of electrons and the high value of the effective mass of holes facilitate accelerating the mobility dynamics of photogenerated electrons onto the surface prior to holes^10^, which may enhance local electron density, benefiting the photo-driving reduction reaction. The centrosymmetry structure of AgInP_2_S_6_ also enables the photoexcited electrons to distribute on the surface of the layer crystal uniformly^11^, which may remarkably reduce the energy barrier for catalytic molecule activation, alter the catalytic reduction pathway, and enhance yield and enrich species of products.

An atomically thin 2D structure is an ideal platform to provide atomic-level insights into the structure-activity relationship^12^. Firstly, the ultrathin structure allows the photo-generated carriers to easily transfer from the interior to the surface with shortened charge transfer distance, decreasing the bulk recombination. Secondly, large surface exposure renders rich catalytic active sites. Thirdly, transparency resulting from ultrathin thickness helps for light absorption. The creation of vacancy defects in the ultrathin structure can also additionally enrich the reaction intermediates, resulting in low-coordinated atoms on the surface of the catalyst, which are known to facilitate to the generation of multi-carbon species from CO_2_ photoreduction^13,14^.

Herein, we report the synthesis of the AgInP_2_S_6_ single atomic layer (abbreviated as SAL) of ~0.70 nm in thickness through a facile probe sonication exfoliation of the corresponding bulk crystal (abbreviated as BC). The sulfur vacancy (abbreviated as V_S_) defects were introduced in the resulting SAL through an etching process with H_2_O_2_ solution (abbreviated as V_S_-SAL), which was prospectively utilized for photocatalytic reduction of CO_2_ in the presence of water vapor. While BC and SAL dominantly produce CO, the implemented defect engineering changes the reaction pathway of the CO_2_ photoreduction on V_S_-SAL, which allows steering CO_2_ conversion into C_2_H_4_ with the yield-based selectivity reaching ~73% and the electron-based selectivity as high as ~89%, and the quantum yield of 0.51% at a wavelength of 415 nm. Both DFT calculation and in situ FTIR spectra demonstrate that the key step for the CO production on BC and SAL follows a conventional hydrogenation process of CO_2_ to form *COOH, which further couples a proton/electron pair to generate *CO. *CO easily liberates from the defect-free AgInP_2_S_6_ surface with low absorption energy to become free CO gas. In contrast, the introduction of V_S_ in AgInP_2_S_6_ causes the charge accumulation on the Ag atoms near V_S_. Thus, the exposed Ag site in V_S_-SAL can effectively capture the forming *CO, making the catalyst surface enrich with key reaction intermediates to promote C–C coupling into C_2_ species with the low binding energy barrier. This work may provide fresh insights into the design of an atomically thin photocatalyst framework for CO_2_ reduction and establish an ideal platform for reaffirming the versatility of defect engineering in tuning catalytic activity and selectivity.

## Results

### Structure characterization of the AgInP_2_S_6_ related samples

BC was synthesized through PVT in a two-zone furnace, which displays bright yellowish-brown color (Supplementary Fig. 1a). The SAL was produced through mechanical exfoliation in ethyl alcohol solution through a probe sonication technique, which can transfer high energy into layered materials and weaken the Van der Waals forces between adjacent layers, resulting in effective delamination. The well-defined Tyndall effect of the resulting transparent solution of SAL indicates high monodispersity of the ultrathin sheets (Supplementary Fig. 1b). Etching of SAL with H_2_O_2_ solutions allows to deliberately create V_S_ on the surface of SAL^15^.

**Fig. 1:**
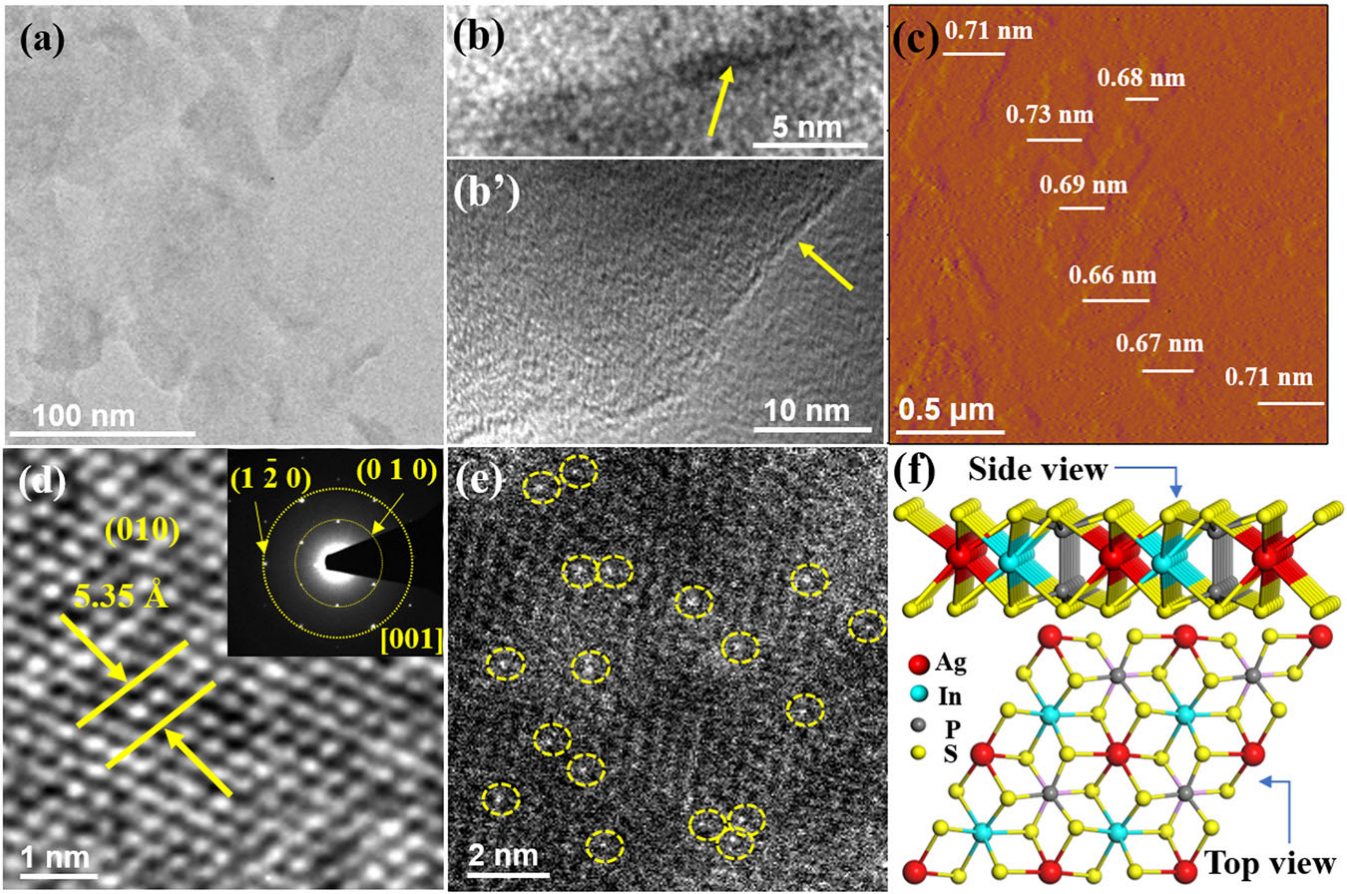
Morphological structure characterization of the fabricated SAL and V_S_-SAL_10_. TEM images of **a** SAL, **b** vertically standing, and **b’** laying single piece SAL, **c** AFM image of SAL showing an average thickness of ~0.69 nm. **d** HRTEM image and the EDS. **e** HAADF-STEM image of V_S_-SAL_10_, in which the atomically dispersed V_s_ are highlighted with the yellow circles. **f** The crystalline models of SAL from top and side views.

The powder X-ray diffraction (XRD) pattern of BC and SAL agrees with the simulated one from the crystal structure of ICSD 202185 well with the ${{{\mbox{P}}}}_{\bar{3}}$_1c_ space group (Supplementary Fig. 2)^12^, and no impurity peaks were detected. The stronger SAL peak intensity ratio of (002) to (112) relative to BC indicates that the exfoliation of AgInP_2_S_6_ occurs along [001] direction. The field emission scanning electron microscopy (FE-SEM) image shows that BC displays an angular shape with an apparent laminar structure (Supplementary Fig. 3a, b). The energy dispersive spectroscopy (EDS) spectra demonstrate the uniform spatial distribution of Ag, In, P, and S (Supplementary Fig. 3c–f). The TEM image of exfoliated SAL displays light contrast of the extremely thin 2D structure (Fig. 1a). A magnified transmission electron microscopy (TEM) image of a vertically standing sheet shows the single layer with a thickness of ∼0.71 nm (Fig. 1b). A typical edge-curling sheet as marked with an arrow also particularly shows the thickness of ~0.72 nm of SAL (Fig. 1b), well in agreement with the AgInP_2_S_6_ monolayer along [002] orientation [*d*_(002)_ = 6.68 Å]. The corresponding atomic force microscopy (AFM) image of SAL also confirms ~0.66–0.73 nm range in thickness (Fig. 1c and Supplementary Fig. 4 for more images), demonstrating the single-atom layer feature. A high-resolution TEM (HRTEM) image of SAL reveals that the interplanar *d*-spacing between the well-defined lattice fringes were examined 0.54 nm, which can be indexed to (010) (Fig. 1d). The selected area electron diffraction shows an ordered array of spots recorded from [001] zone axis (Fig. 1d, inset), confirming that SAL is of single crystallinity and preferentially enclosed by {002} top and bottom surfaces. The crystalline model of SAL from top and side views was schematically illuminated in Fig. 1f. With H_2_O_2_ solution treatment for optimized 10 s, the sulfur atoms, which locate outermost in SAL, can be partially etched away from the surface to form V_S_. The generation of V_S_ was confirmed with the electron paramagnetic resonance (EPR) spectra (Supplementary Fig. 5). The Raman spectra show that the peak intensity of both S–P–P and P–S–P for Vs-SAL were lowered, compared with those of SAL (Supplementary Fig. 6), which additionally verifies the detected defect sites can be assigned to V_S_^16^, rather than the open of S–M (metal) bond or the possible insertion of O atoms.

No obvious difference of the XRD patterns between SAL and Vs-SAL demonstrates no crystal structure change of the SAL before and after H_2_O_2_ etching treatment (Supplementary Fig. 2). The TEM image also shows that the resulting V_S_-SAL_10_ displays no morphology change in ultrathin structure (Supplementary Fig. 7). The corresponding EDS reveals that Ag, In, and P contents were nearly stoichiometric 1:1:2 of AgInP_2_S_6_, expect S element less than the stoichiometric ratio (Supplementary Fig. 8). It indicates that H_2_O_2_ treatment mainly leads to V_S_, and has no etching effect on other moieties, which was also verified with the following XPS and the X-ray absorption near edge structure (XANES) spectra. The atomic resolution, aberration-corrected high-angle annular dark-field scanning TEM (HAADF-STEM) clearly reveals that a considerable number of V_S_ were confined in the sheet (Fig. 1e), in contrast to the few sporadic ones in SAL (Supplementary Fig. 9).

**Fig. 2:**
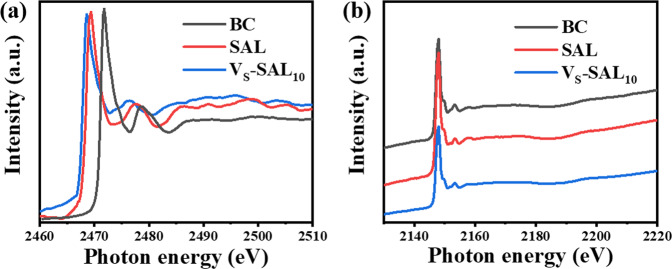
XANES spectra of BC, SAL, and Vs-SAL10. **a** S and **b** P K-edge XANES spectra of BC, SAL, and Vs-SAL10.

Full XPS spectra demonstrate the presence of Ag, In, P, and S (Supplementary Fig. 10a). The high-resolution S *2p* spectrum of BC shows the S *2p* peak falling between 162 and 164 eV (Supplementary Fig. 10b), revealing the −2 oxidation state of S. The S 2*p* peaks of SAL show dramatic low binding energy shift, compared with BC, and V_S_-SAL_10_ possesses further low-energy shift. The former shift may originate from exfoliation-resulting monolayerization^17^ and the latter from V_S_^15^. As the decrease of binding energy indicates the enhanced electron screening effect due to the increase of the electron concentration^15,18^, it implies that the electron density around the S sites increases in the sequence of BC, SAL, and V_S_-SAL_10_. It reveals that the residual S atoms exist in an electron oversaturated form and possess high electron density. No obvious change of binding energy of P elements was observed (Supplementary Fig. 10c), further demonstrating that the mechanical exfoliation and chemical etching only damage sulfur atoms and have little effect on P moiety. Weak O*1s* XPS peaks were observed for both SAL and Vs-SAL_10_ (Supplementary Fig. 10d), which more likely originate from absorbed components from the ambiance. The almost same intensity and location of O*1s* peak indicate no apparent oxidation change before and after H_2_O_2_ treatment. The pre-edge characteristic of the XANES spectra of the S K-edges of three AgInP_2_S_6_ was shown in Fig. 2a, which could be fitted with components of a spin−orbit split. The spectra indicate the existence of main transitions energies between 2460 and 2500 eV, which originates from the excitation of an electron from a 1S inner orbital to a higher-energy orbital as a result of interaction with an X-ray. In comparison with BC, SAL shows a shift for S K-edge peaks to the lower energy side. This can be explained by the fact that the core electrons of S become more loosely bound after mechanical exfoliation due to the increased screening of the nuclear charge. Through V_S_ engineering, the S K-edge of V_S_-SAL_10_ can have a further small move to the lower energy side (Fig. 2a). Moreover, the K-edge peak of P between 2100 to 2250 eV exhibits almost no differences among BC, SAL, and V_S_-SAL_10_ (Fig. 2b), which is in good agreement with the above-mentioned XPS results.

The UV–vis diffuse reflectance spectra show that the bandgap of SAL was determined 2.66 eV, a little larger than that of BC (2.31 eV) (Supplementary Fig. 11), exhibiting a strong quantum size effect in the lateral direction. V_S_-SAL_10_ displays a slightly narrowed bandgap (2.57 eV) with respect to SAL. It derives from that introduction of V_S_ may tailor the electronic structure of SAL through generating impurity states near the conduction band (CB) edge, which can be overlapped and delocalized with the CB minimum edge, leading to a reduced bandgap that may broaden the light absorption edge^19,20^. The XPS spectra show that the Ag_*3d*_ peak of Vs-SAL_10_ shifts to lower binding energy relative to that of SAL (Supplementary Fig. 10e), confirming the valance changes of Ag in Vs-SAL_10_. The VB change of AgInP_2_S_6_ may lead to the corresponding changes of its CB^20^. The Mott–Schottky plots reveal that the CB edge of V_S_-SAL_10_ upshifts by ~0.06 and ~0.26 eV, relative to that of SAL and BC, respectively, as schematically illustrated in Supplementary Fig. 12. All BC, SAL, and V_S_-SAL_10_ were thus confirmed to possess suitable bandgaps as well as the appropriate band edge positions for photocatalytic CO_2_ reduction under visible-light irradiation.

### Photocatalytic performance toward CO_2_ photoreduction

**Fig. 3:**
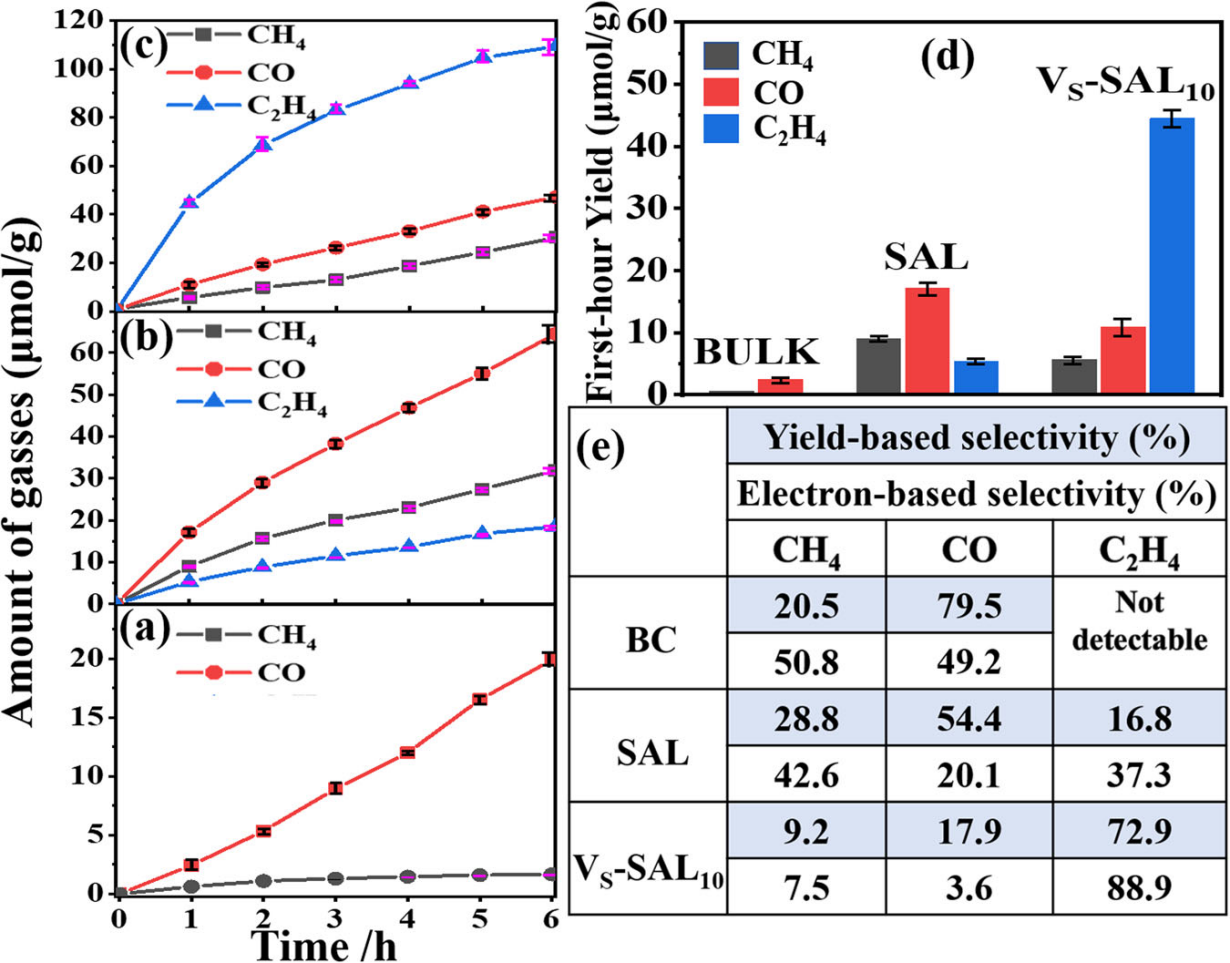
Photocatalytic CO_2_ reduction performance. Photocatalytic gases evolution amounts as a function of light irradiation times of **a** BC, **b** SAL, and **c** V_S_-SAL. **d** Photocatalytic activity for the first hour. **e** Table illustration for the yield and electron-based selectivities of photocatalytic CO_2_ conversion.

The photocatalytic CO_2_ conversion was carried out in the presence of water vapor under simulated solar irradiation (Fig. 3). CO was detected the major product for BC and SAL (Fig. 3a, b). BC shows the CO yield of 2.44 μmol g^−1^ for the first hour and a trace amount of CH_4_ of 0.63 μmol g^−1^ (Fig. 3a). The photogenerated holes in the VB oxidize H_2_O to produce hydrogen ions by the reaction of H_2_O → 1/2O_2_ + 2H^+^ + 2e^−^. CO is formed by reacting with two protons and two electrons (${{{{\rm{CO}}}}}_{2}+{{{{\rm{2e}}}}}^{-}+{{{{\rm{2H}}}}}^{+}\to {{{\rm{CO}}}}+{{{{\rm{H}}}}}_{2}{{{\rm{O}}}}\,{{(1)}}$), and CH_4_ formation through accepting eight electrons and eight protons (${{{{\rm{CO}}}}}_{2}+{{{{\rm{8e}}}}}^{-}+{{{{\rm{8H}}}}}^{+}\to {{{{\rm{CH}}}}}_{4}+{{{{\rm{2H}}}}}_{2}{{{\rm{O}}}}\,{{(2)}}$). SAL exhibits 6.9 and 14.3-time enhancement of production of CO and CH_4_ relative to BC, reaching 17.1 and 9.0 μmol g^−1^ for the first hour, respectively (Fig. 3b). A small amount of H_2_ was also generated as a typical competitive reaction with CO_2_ reduction (Supplementary Fig. 13). The prerogative of atomic ultrathin geometry of SAL may be mainly responsible for the enhanced photocatalytic activity besides larger surface area, allowing charge carriers to move from interior to the surface quickly to conduct catalysis, avoiding the recombination in the body. A small amount of C_2_H_4_ was also detected for SAL with a yield of 5.3 μmol g^−1^. C_2_H_4_ is generated by accepting 12 electrons and 12 protons (${{{{\rm{2CO}}}}}_{2}+{{{{\rm{12e}}}}}^{-}+{{{{\rm{12H}}}}}^{+}\to {{{{\rm{C}}}}}_{2}{{{{\rm{H}}}}}_{4}+{{{{\rm{4H}}}}}_{2}{{{\rm{O}}}}\,{{(3)}}$). With the H_2_O_2_ etching process, excitingly, C_2_H_4_ excitingly becomes the main product for V_S_-SAL_10_ with a yield of 44.3 μmol g^−1^ (Fig. 3c). The calculated yield-based selectivity reaches ~73%, and the electron-based selectivity is as high as ~89%^21^ (Fig. 3e). Meanwhile, CO and CH_4_ minority products were also traced with the yields of 10.9 and 5.6 μmol g^−1^, respectively, both less than the case of SAL. It indicates that the surface of V_S_-SAL_10_ preferentially promotes the C_1_ intermediates to C–C couple into C_2_ product rather than liberate them into free CO and CH_4_ gases. The quantum yield of V_S_-SAL_10_ was measured 0.51% at a wavelength of 415 nm using monochromatic light (see the details in SI). The etching process time was found determinative for the dominant production of C_2_H_4_. The EPR measurement shows that the signal intensity gradually increases with prolonging etching time from 5 to 15 s (Supplementary Fig. 5), indicating being raised a number of V_S_ in V_S_-SAL. Elongation of the etching time from 5 to 10 s was favorable for increasing the yield of C_2_H_4_ (Supplementary Fig. 14). However, a much long etching time of 15 s decreases activity negatively, which may be due to that an excess of V_S_ defects may accelerate the recombination of photogenerated carriers^22^. The reduction experiment of CO_2_ performed in the dark or absence of the photocatalyst shows no appearance of CO and hydrocarbon products, proving that the reduction reaction of CO_2_ is driven by light under photocatalyst. A blank experiment with the identical condition and in the absence of CO_2_ shows no appearance of C_2_H_4_, CO, and CH_4_, proving that the carbon source was completely derived from input CO_2_. An isotope labeling experiment using ^13^CO_2_ confirms that the produced C_2_H_4_ originates from the input CO_2_ (Supplementary Fig. 15). The O_2_ production was also detected using the similar isotope H_2_^18^O tracer control experiment (Supplementary Fig. 15). It should be mentioned that after 12 h light irradiation, increased tendency of the generation of the hydrocarbon products over the present photocatalyst slowed down. It may be assigned to the potential carbon deposition as intermediates covering the active sites of the photocatalyst during the photoreduction process. The problem may be resolved through post washing treatment to recover the catalytic activity to a certain extent, as shown in Fig. S16.

### Mechanism of the photocatalytic performance of the V_S_-SAL

**Fig. 4:**
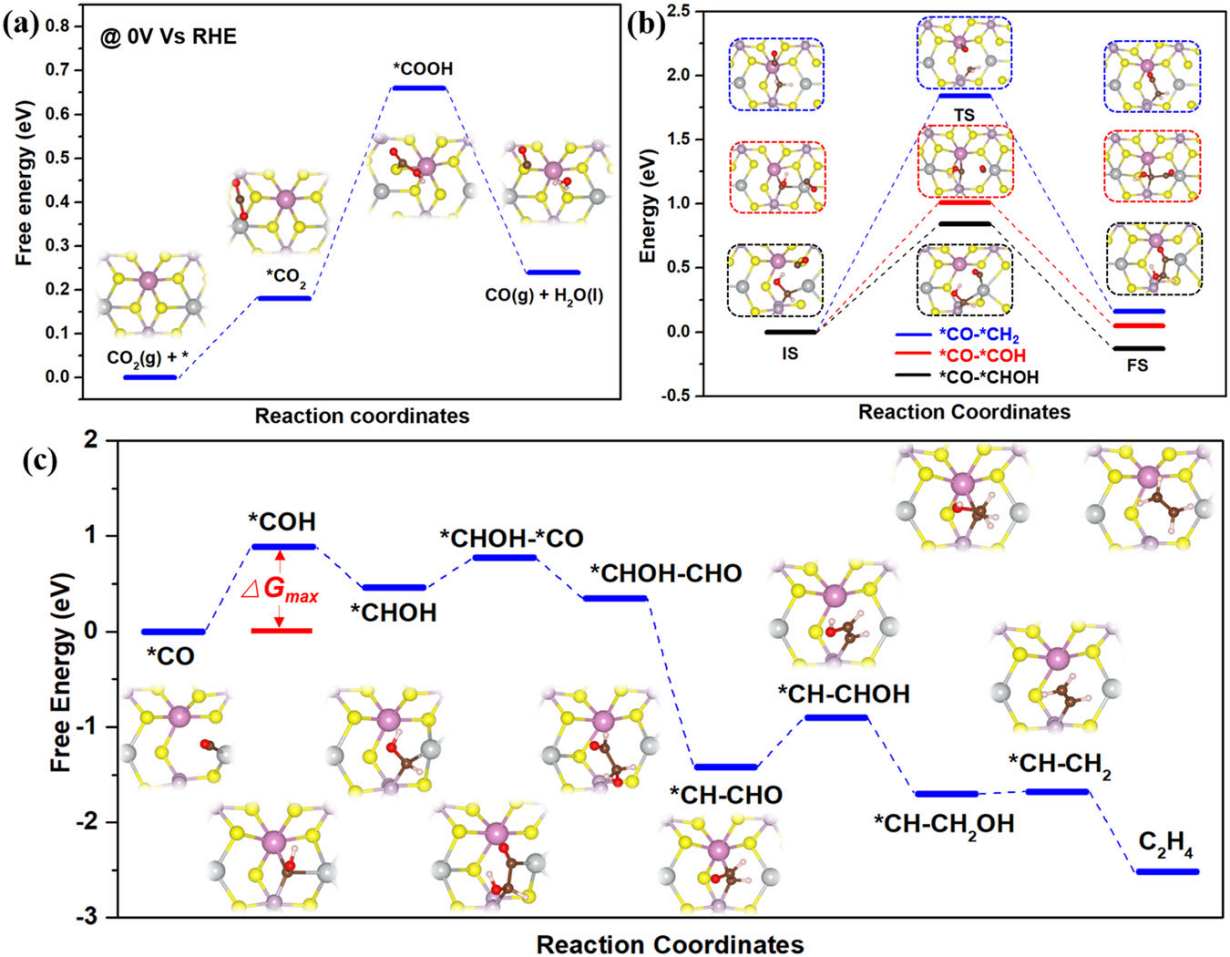
Theoretical investigations. **a** Gibbs free energy diagrams for CO_2_ reduction to CO over perfect AgInP_2_S_6_. **b** Three kinds of possible C–C coupling pathways over AgInP_2_S_6_ containing V_s_. **c** Gibbs free energy diagrams for CO reduction to C_2_H_4_ over AgInP_2_S_6_ with V_s_. The insets show the corresponding optimized geometries for the reaction intermediates during the CO_2_ reduction process. Sulfur, phosphorus, indium, silver, carbon, oxygen, and hydrogen atoms are yellow, purple, lilac, gray, black, red, and white, respectively.

DFT simulations were performed to explore the V_S_-mediated catalytic selectivity mechanism toward CO and C_2_H_4_ on AgInP_2_S_6_. CO_2_ molecules are initially adsorbed on the catalyst surface where H_2_O molecules dissociate into hydroxyl and hydrogen ions at the same time. The free-energy profile for the photocatalytic CO_2_-to-hydrocarbon process with the lowest-energy pathway on the perfect AgInP_2_S_6_ surface was calculated, as shown in Fig. 4. The key step for CO production is the hydrogenation of CO_2_ to form *COOH, and the free-energy change of the step is 0.48 eV. Subsequently, the reaction intermediate (*COOH) further couples a proton/electron pair to generate CO and H_2_O molecules. The adsorption energy of −0.07 eV of the produced *CO on the defect-free AgInP_2_S_6_ surface implies the physical adsorption on the catalyst (Supplementary Fig. 17a). It means that *CO molecules can easily liberate from BC and SAL to become free CO gas, allowing high CO catalytic selectivity. Additional parts of *CO were continuously reduced by the incoming electrons and the successive protonation process to transform into CH_4_^20,23^. While the charge density of the valence band (VB) for pristine AgInP_2_S_6_ is evenly located on all the S and Ag atoms, contrastingly, the charge density of the VB is mainly located on the Ag atoms near the V_S_ for V_S_-AgInP_2_S_6_, (Supplementary Fig. 18). That is to say, the presence of V_S_ in V_S_-AgInP_2_S_6_ causes the charge enrichment on the Ag atoms near the V_S_, which would benefit for stabilizing the reaction intermediates. For V_S_-SAL, V_S_ can act as a trap for the *CO molecule, that is, the *CO molecule can chemically adsorb at exposed Ag sites with an adsorption energy of −0.25 eV (CO can only physically adsorb on the exposed P and In sites with a distance of 2.56 and 3.20 Å, See Supplementary Fig. 17b–d). The higher CO onset desorption temperature on V_S_-SAL_10_ than SAL affirms the stronger absorption (Supplementary Fig. 19). The absorbed *CO can be further protonated to successively form a series of key reaction intermediates with unsaturated coordination, which was confirmed with in situ FTIR measurement (Supplementary Fig. 20). The other *CO molecules produced on the surface diffuses toward V_S_ and couple with those reaction intermediates to produce C_2_H_4_. The C_2_H_4_ free energy diagrams are summarized in Fig. 4c, while the corresponding C–C coupling barriers are presented in Fig. 4b. The different C-C coupling energy barriers were evaluated for three unsaturated reaction intermediates (*COH, *CHOH, and *CH_2_) (Fig. 4b). The coupling energy barrier with a value of 0.84 eV (*CO–CHOH) is lower than that of other coupling pathways (*CO–COH, 1.01 eV and *CO–CH_2_, 1.84 eV), hence the C_2_H_4_ will be produced via CO–CHOH coupling and hydrogenation. The whole free energy diagram shows that the process of *CO to *COH is regarded as the potential determining step (0.86 eV). It should be especially emphasized that the detected small amount of C_2_H_4_ on SAL possibly originates from the potential existence of the tiny number of V_S_ in SAL, resulting from mechanically detaching sulfur atoms from SAL during the probe sonication exfoliation process. The reaction process for the reduction of CO_2_ into C_2_H_4_, CO, and CH_4_ over V_S_-SAL under light illumination is thus proposed in Supplementary Fig. 21. To confirm CO as an important intermediate for the C_2_H_4_ formation, CO as the starting reactant substituting for CO_2_ was also conducted for the similar photocatalytic performance. The result reveals that a considerable amount of C_2_H_4_ was indeed detected (Supplementary Fig. 22). In addition, a small amount of ethane (C_2_H_6_) and propylene (C_3_H_6_) were also produced. It indicates that CO as starting reactants may be further favorable for C–C, even C_2_–C coupling.

**Fig. 5:**
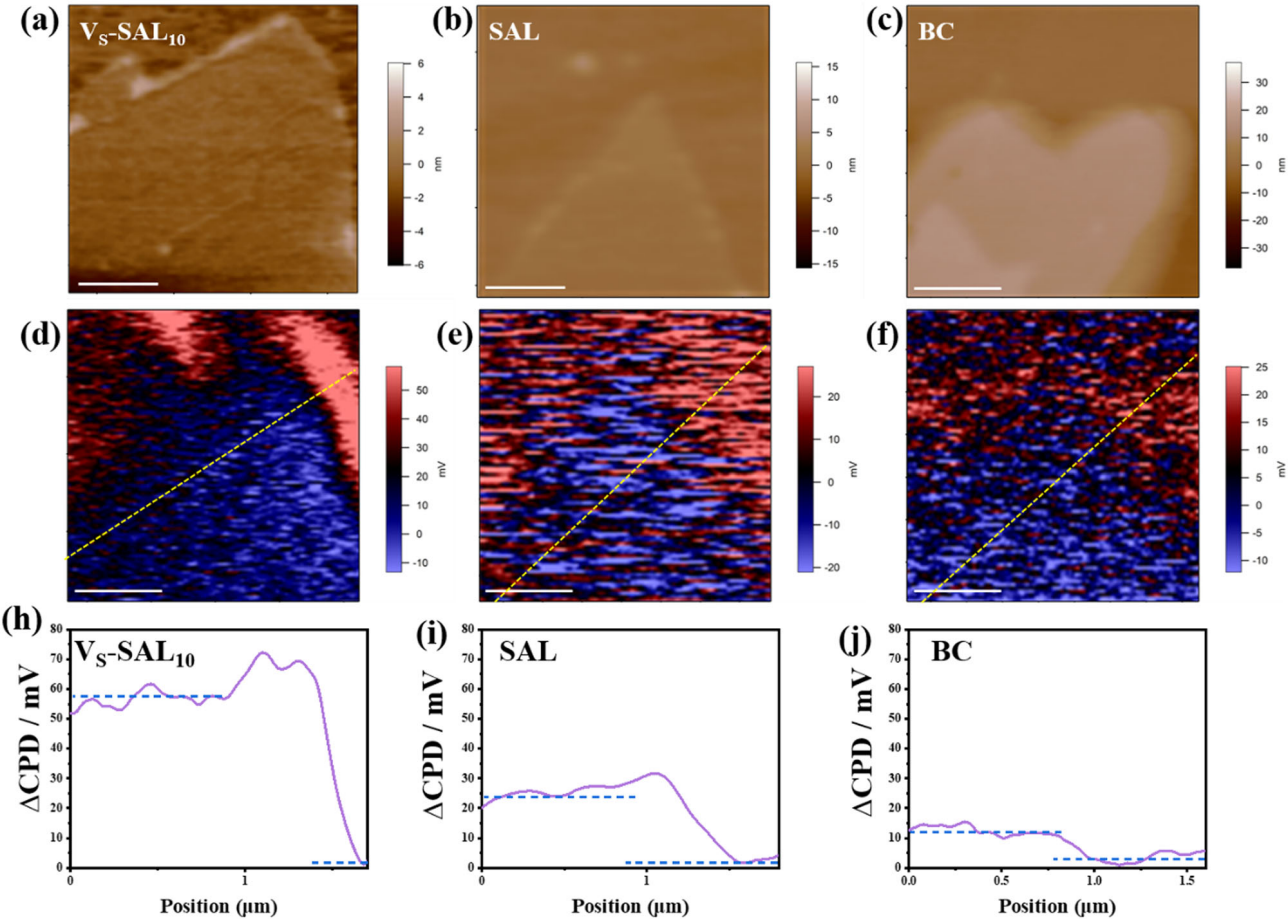
SPV characterization. Height images of **a** V_S_-SAL_10_, **b** SAL, and **c** BC. The SPV images **d** V_S_-SAL_10_, **e** SAL, and **f** BC in (**a**–**c**), respectively, are differential images between potential images under light and in the dark. All scale bars represent 0.5 μm. The surface photovoltage change by subtracting the potential under dark conditions from that under illumination (SPV, ΔCPD = CPD dark − CPD light) of **h** V_S_-SAL_10_, **i** SAL, and **j** BC.

Surface photovoltage spectroscopy (SPV) was employed to study the separation and transport behavior of photoinduced charge carriers of the studied AgInP_2_S_6_. More negative SPV signal change reflects a higher concentration of photogenerated electrons before and after light illumination. All BC, SAL, and Vs-SAL_10_ show the SPV response under light illumination (Fig. 5 and Supplementary Fig. 23), corresponding to band-to-band transition. The SAL and BC exhibit 20–30 mV and 5–10 mV negative change before and after light illumination, respectively. More negative SPV signal change of SAL than BC exactly demonstrates that the atomically thin structure enables to alleviate the bulk electron–hole recombination to achieve high-concentration accumulation of photogenerated electrons on the surface. The V_S_-SAL_10_ display obviously dramatic change of 50–60 mV, indicating that introduction of V_S_ can further favor the carrier separation and allow much increment of electron concentration on the surface. The excess surviving electrons are not only the necessary prerequisite to photoconversion of CO_2_, but also can promote CO_2_ adsorption and activation on the surface of the photocatalyst.

Photoluminescence (PL) decay profiles show that the SAL (~1.32 ns) possesses a longer PL lifetime than BC (~0.40 ns) (Supplementary Fig. 24), demonstrating that the atomically thin structure can indeed shorten the transfer distance of the carriers and decrease recombination chance of electron and hole in the body. V_S_-SAL_10_ exhibits the longest PL lifetime (~1.50 ns), confirming that the surface V_S_ can serve as surface separation centers for charge carriers and further promote the charge separation, therefore offering more opportunities for photocatalytic CO_2_ reduction. Transient photocurrent shows that the photocurrent intensity of SAL was enhanced with a steadily repeating course due to promoted charge separation, compared with BC (Supplementary Fig. 25a). The highest photocurrent intensity of V_S_-SAL_10_ implies that the V_S_ also makes an effective contribution to saving carriers. Electrochemical impedance spectra reveal that V_S_-SAL_10_ manifests the smallest semicircle in Nyquist plots (Supplementary Fig. 25b), suggesting the lowest charge-transfer resistance, which permits fast transport of photoinduced charge.

## Discussion

In summary, single atomically thin AgInP_2_S_6_ layers were successfully synthesized through a facile probe sonication exfoliation of BC. The atomically thin structure of SAL, relative to BC, enables more charge carriers to mobile from the interior onto the surface and surviving accumulate onto the active sites to improve the photocatalytic activity. While SAL exhibits obvious conversion efficiency with CO as the major product, the presence of V_S_ in V_S_-SAL changes the CO_2_ photoreduction pathway to allow the dominant generation of C_2_H_4_. This work not only paves an effective approach for selectively producing multi-carbon products from CO_2_ photoreduction but also provides a new insight for catalyst design through vacancy defect engineering.

## Methods

### Synthesis of BC, SAL, and V_S_-SAL

The AgInP_2_S_6_ crystals have been synthesized by physical vapor transport (PVT) in a two-zone furnace. Stoichiometric amounts of high-purity elements (mole ratio Ag: In: P: S = 1:1:2:6, around 1 g in total) were sealed into a quartz ampoule with the pressure of 1 × 10^−4^ Torr inside the ampoule. The length of the quartz ampoule was about 15–18 cm with a 13 mm external diameter. The ampoule was kept in a two-zone furnace (680 → 600 °C) for 1 week^11^. After the furnace was cooled down to room temperature, the AgInP_2_S_6_ crystalline powders could be found inside the ampoule (Supplementary Fig. 1a). SAL was prepared by sonication-assisted liquid exfoliation processes from synthetic AgInP_2_S_6_ crystalline powders. For the detail, AgInP_2_S_6_ bulk powder was ground carefully, and then dispersed in ethanol solution. After continuous ultrasonification for 12 h with a probe-type cell crusher (~1200 W), the solution was conducted for static settlement, and the supernatant was taken to ultrasonic dissection further. Through 4000 × *g* for 10 min centrifugation, the samples peeled insufficiently were removed, and the supernatant was collected through an additional 12,000 × *g* for 20 min centrifugation to obtain the AgInP_2_S_6_ monolayer. The derived SAL has dispersed in the water again for subsequent liquid nitrogen refrigeration and being dried in a vacuum freezing dryer at a pressure below 20 Pa for 2 days. The residual ethanol can be considered to be totally removed.

SAL was immersed in H_2_O_2_ solutions with the of concentrations 0.1 mol/L inside which SAL was allowed to react with H_2_O_2_ for 5, 10, and 15 s, referred to V_S_-SAL_5_, V_S_-SAL_10_, and V_S_-SAL_15_, respectively, at 25 °C. All the obtained samples were carefully washed and dried before use.

### Characterizations

XRD (Rigaku Ultima III, Japan) was used to investigate the purity information and crystallographic phase of the as-prepared powder samples. The XRD pattern was recorded by using Cu-ka radiation (*λ* = 0.154178 nm) at 40 kV and 40 mA with a scan rate of 10° min^−1^. The morphology was characterized by the FESEM (FEI NOVA NANOSEM 230). The TEM and HRTEM images were taken on a JEM 200CX TEM apparatus. X-ray photoelectron spectroscopy (XPS; K-Alpha, Thermo Fisher Scientific) was standardized according to the binding energy of the adventitious C 1s peak at 284.8 eV, which was used to inspect the chemical states. A UV–vis spectrophotometer (UV-2550, Shimadzu) was hired to record the UV-visible diffuse reflectance spectra and switched to the absorption spectrum on the basis of the Kubelka−Munk connection at room temperature. In situ FTIR spectra were measured with synchronous illumination Fourier transform infrared spectroscopy on Bruker IFS 66V FT spectrometer. The PL decay profile was described by the single-particle confocal fluorescence spectroscopy measurement (PicoHarp300). SPV was detected through AFM (Asylum Research, MFP-3D-SA, USA) analysis with the photo-assisted (a 405 nm laser excitation) Kelvin probe force microscopy. Photoelectrochemical measurements were detected by a CHI660E electrochemical workstation using a standard three-electrode system in 1 mM NaSO_4_ solution. Soft X-ray absorption spectra (XAS) were collected from the Soft X-ray Spectroscopy beamline at the Australian Synchrotron (AS, Australia), part of ANSTO.

For the electrochemistry measurement, the AgInP_2_S_6_ catalyst ink was prepared by dispersing 10 mg of as-prepared catalysts in 1 mL of ethanol under sonication. Then, 50 μL of the ink was evenly spread onto a piece of pretreated FTO within a 1 cm^2^ area and dried at room temperature. The catalysts were thus attached to FTO. The solid-state current–voltage (*J*–*V*) test curves exhibit Ohmic characteristics (Supplementary Fig. 26), confirming the formation of ohmic back contact between samples and FTO. The working area of the electrode is as large as 1 cm^2^. The scan rate was 5 mV s^−1^. The reference electrode was the saturated Ag/AgCl electrode, and a Pt foil was employed as the counter electrode. The 0.5 M Na_2_SO_4_ aqueous solution was used as the electrolyte.

### Measurement of photocatalytic activity

For the photocatalytic reduction of CO_2_, 4–5 mg of sample was uniformly dispersed on the glass reactor with an area of 4.2 cm^2^. A 300 W Xenon arc lamp was used as the light source of the photocatalytic reaction. The volume of the reaction system was about 460 ml. Before the irradiation, the system was vacuum-treated several times, and then the high purity of CO_2_ gas was followed into the reaction setup for reaching ambient pressure. Totally, 0.4 mL of deionized water was injected into the reaction system as a reducer. The as-prepared photocatalysts were allowed to equilibrate in the CO_2_/H_2_O atmosphere for several hours to ensure that the adsorption of gas molecules was complete. During the irradiation, about 1 mL of gas was continually taken from the reaction cell at given time intervals for subsequent CO, CH_4_, and C_2_H_4_ concentration analysis by using a gas chromatograph (GC-2014C, Shimadzu Corp., Japan).

### The external quantum efficiency (EQE)

The quantum yield was calculated according to the below equation

4 $${{E}}_{{{{\rm{Q}}}}}=	\, {N}\,({{{\rm{electron}}}})/{N}\,({{{\rm{photon}}}})\\ =	\, [{N}\,({{{\rm{CO}}}})\times 2+{N}\,({{{{\rm{CH}}}}}_{4})\times 8+{N}\,({{{{\rm{C}}}}}_{2}{{{{\rm{H}}}}}_{4})\,\times 12]/{N}\,({{{\rm{photon}}}})\,\times 100 \%$$

where *N* (electron) signifies two electrons are required to produce one molecule CO in unit time. The *N* (photon) is figured out according to the equation:

5 $${N}\,({{{\rm{photon}}}})=[{{{\rm{light}}}}\,{{{\rm{intensity}}}}\times {{{\rm{illumination}}}}\,{{{\rm{area}}}}\times {{{\rm{time}}}}]/[{{{\rm{average}}}}\,{{{\rm{single}}}}\,{{{\rm{photon}}}}\,{{{{\rm{energy}}}}\times {N}}_{{{{\rm{A}}}}}]$$

Light-emitting diodes (LEDs) provides the monochromatic incident light with identical conditions. The light intensity of LEDs with 415 nm wavelength is 10.5 mW/cm^2^, the illumination area is controlled to 4.91 cm^2^, *N*_A_ is the Avogadro constant, and the average single photon energy is calculated according to the equation:

6 $${E}\,({{{\rm{photon}}}})={hc}/{\lambda}$$

in which *h* is the Planck constant, c indicates the speed of light, and *λ* is the wavelength.

### Computational details

The density functional theory (DFT) calculations were made with the Vienna Ab Initio Simulation Package^24,25^ code. The exchange-correlation interactions and the ion–electron interactions were solved by the Perdew–Burke–Ernzerhof functionals^26,27^ and the projector-augmented wave method^28^, respectively. The monolayer AgInP_2_S_6_ was a model with a 2 × 2 supercell. A plane-wave cutoff of 450 eV was adopted and the maximal force on all-atom was below 0.02 eV/Å. The distance between periodic units in the vertical direction was larger than 16 Å. The DFT-D2 method of Grimme^29^ was used in all calculations to accurately describe long-range Van der Waals (vdW) interactions. The climbing-image nudged elastic band (CI-NEB) method^30^ incorporated with spin-polarized DFT was used to locate the minimum-energy path. The intermediate images of each CI-NEB simulation were relaxed until the perpendicular forces were smaller than 0.1 eV/Å.

The free energies of each reaction intermediates were determined according to *G* = *E* + ZPE − TS. (7) The electronic energies (*E*) can be directly obtained from DFT computations. The zero-point energy (ZPE) and entropy correction (TS) were calculated from vibration analysis by standard methods. The computational hydrogen electrode model^31^ was used to treat the free energy change of each reaction step involving a proton–electron pair transfer. In this model, the free energy of a proton-electron pair at 0 V vs. RHE is equal to half of the free energy of a hydrogen molecule.

## Supplementary information

Supplementary Information

Peer Review File

## Data Availability

The data that support the findings of this study are available from the corresponding author upon reasonable request.
