# Supplementary material for: Vacancy-defect modulated pathway of photoreduction of CO2 on single atomically thin AgInP2S6 sheets into olefiant gas
Source: Nat Commun. 2021 Aug 6;12:4747. doi: 10.1038/s41467-021-25068-7 (PMC8346554; doi:10.1038/s41467-021-25068-7)
Supplement: Supplementary file 1 — Supplementary Information [file 41467_2021_25068_MOESM1_ESM.pdf]

# Supporting Information

## Vacancy-Defect Modulated Pathway of Photoreduction of $CO_2$ on Single Atomically Thin $AgInP_2S_6$ Sheets into Olefiant Gas

Wa Gao,[1,‡] Shi Li,[3,‡] Huichao He,[2] Xiaoning Li,[5] Zhenxiang Cheng,[5] Yong Yang,[6] Jinlan Wang,*[,3] Qing Shen,[7] Xiaoyong Wang,[1] Yujie Xiong,*[,4] Yong Zhou,*[,1,8] Zhigang Zou[1,8]

[1] Key Laboratory of Modern Acoustics (MOE), Institute of Acoustics, School of Physics, Jiangsu Key Laboratory of Nanotechnology, Eco-materials and Renewable Energy Research Center (ERERC), National Laboratory of Solid State Microstructures, Collaborative Innovation Center of Advanced Microstructures, Nanjing University, Nanjing 210093, China

[2] State Key Laboratory of Environmental Friendly Energy Materials, Southwest University of Science and Technology, Mianyang 621010, China

[3] School of Physics, Southeast University, Nanjing 211189, China

[4] Hefei National Laboratory for Physical Sciences at the Microscale, Collaborative Innovation Center of Chemistry for Energy Materials (iChEM), School of Chemistry and Materials Science, University of Science and Technology of China, Hefei, Anhui 230026, China

[5] Institute of Superconducting & Electronic Materials, Innovation Campus, University of Wollongong, Squires Way, North Wollongong, NSW 2500, Australia

[6] Key Laboratory of Soft Chemistry and Functional Materials (MOE), Nanjing University of Science and Technology, Nanjing 210094, China

[7] University of Electrocommunication, Grad Sch Informatics and Engineering, 1-5-1 Chofugaoka, Chofu, Tokyo 1828585, Japan.

[8] School of Science and Engineering, The Chinese University of Hongkong (Shenzhen), Shenzhen, Guangdong 518172, China

‡Gao and Li contributed to the work equally.

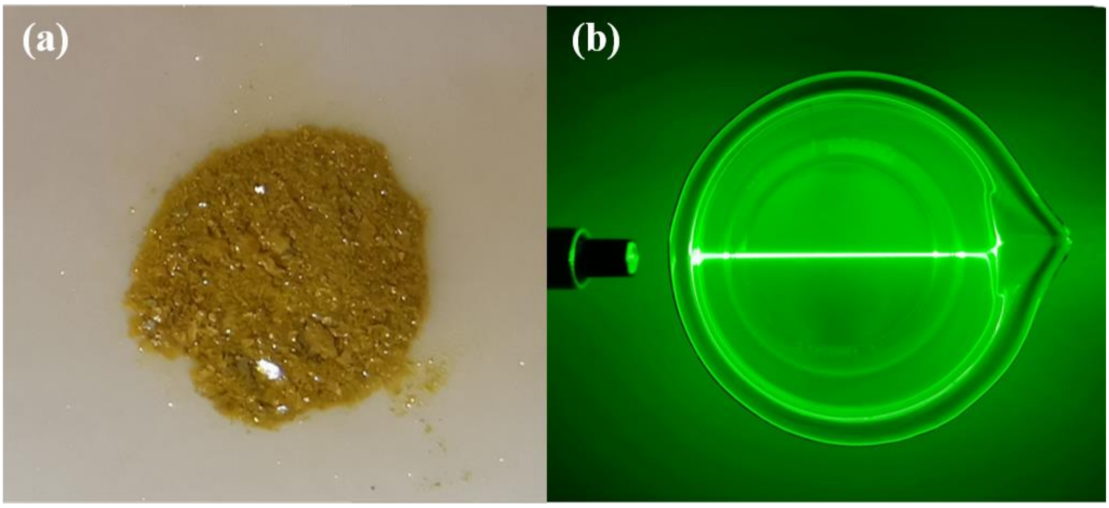


**Supplementary Figure 1.** Pictures of the synthesized $AgInP_2S_6$. (a) Picture of BC powder, and (b) Corresponding colloidal suspension of SAL. A laser beam was incident from the left side to demonstrate Tyndall effect of SAL solution.

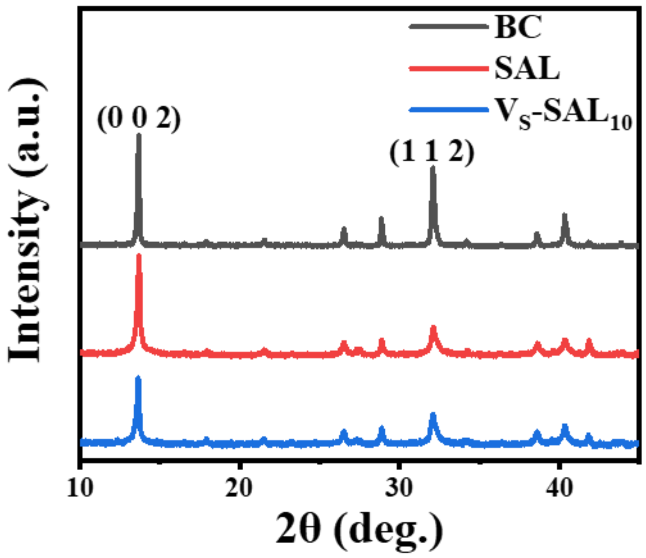


**Supplementary Figure 2.** XRD patterns of BC, SAL, and $V_S$-SAL$_{10}$.

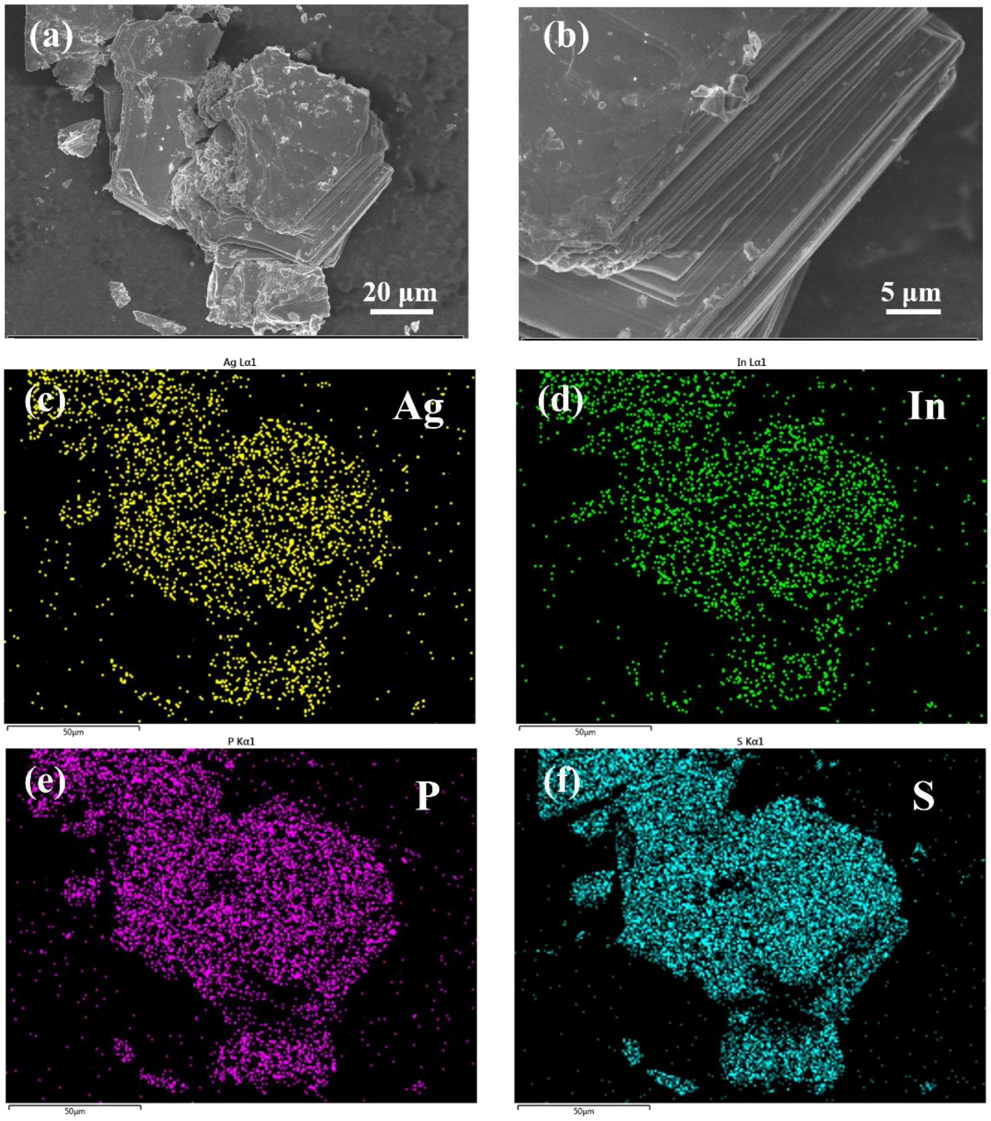


**Supplementary Figure 3.** Morphological structure characterization of BC. (a, b) SEM images of BC. (c-f) EDS spectra of Ag, In, P, and S of BC.

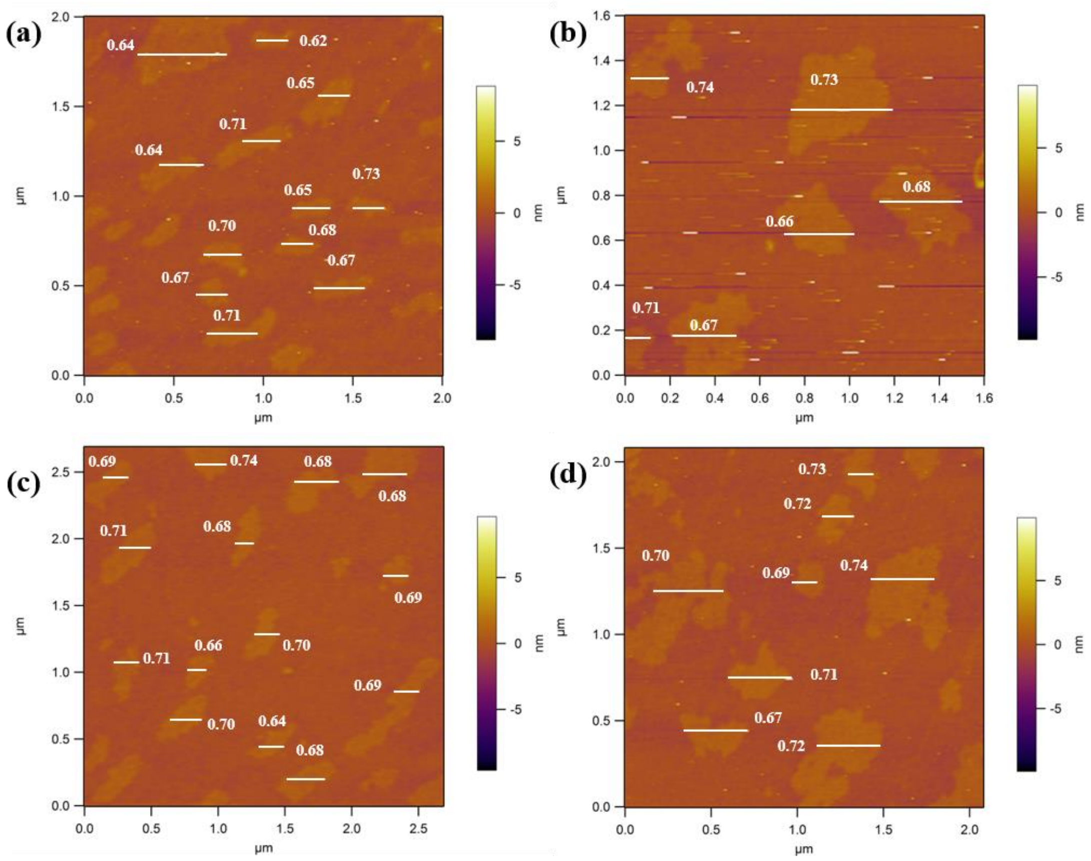


**Supplementary Figure 4.** AFM image of SAL.

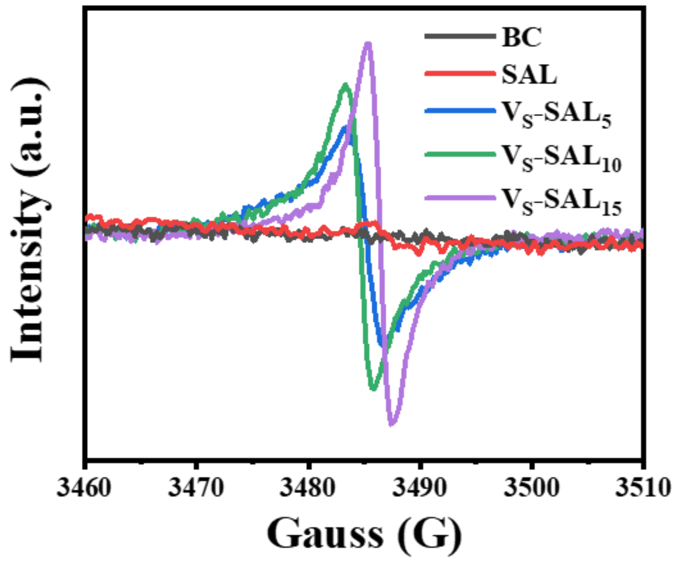


**Supplementary Figure 5.** EPR spectra of BC, SAL, and $H_2O_2$-treating $AgInP_2S_6$ with different etching time.

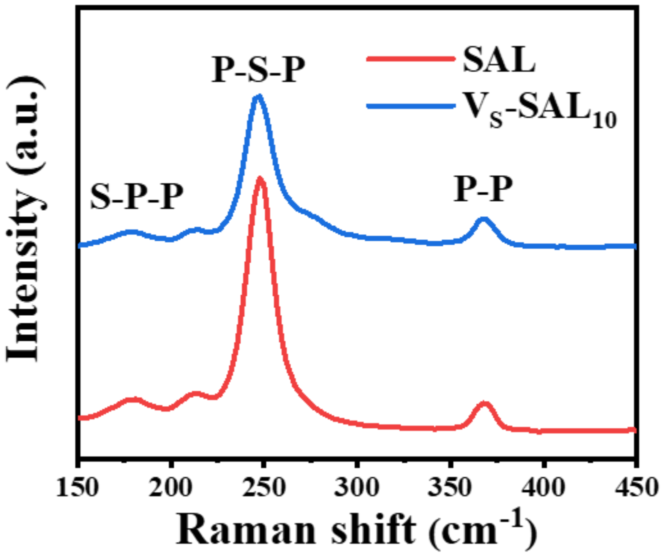


**Supplementary Figure 6.** Raman spectra of $V_S$-SAL and SAL.

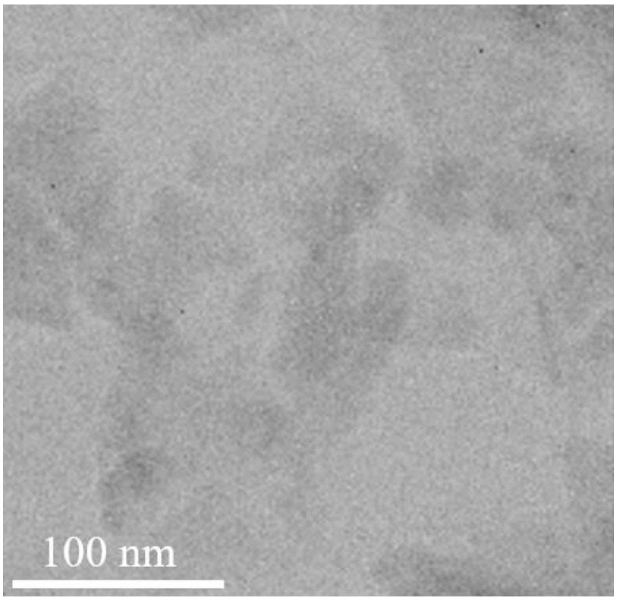


**Supplementary Figure 7.** TEM image of $V_S$-$SAL_{10}$.

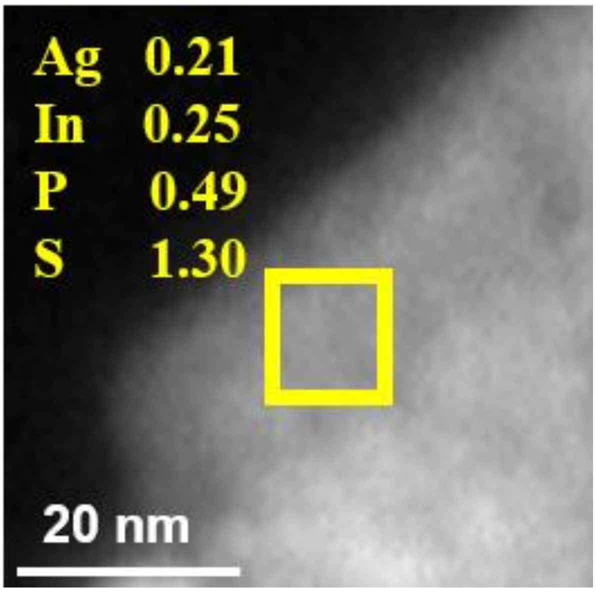


**Supplementary Figure 8.** EDS elemental analysis of $V_S$-$SAL_{10}$ of selected-area in the image.

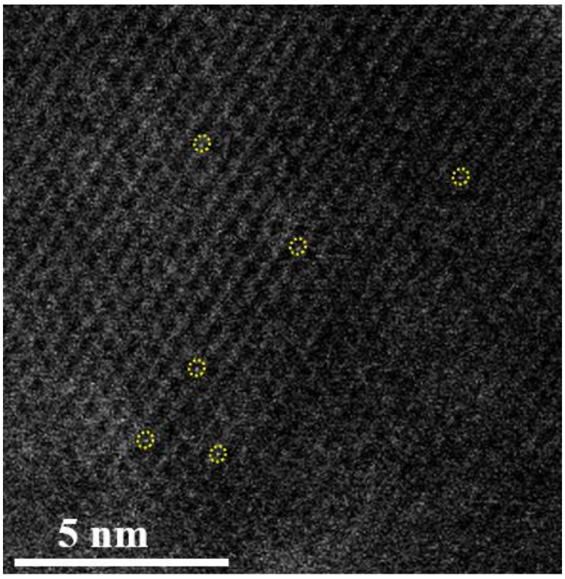


**Supplementary Figure 9.** HAADF-STEM images of the parent SAL.

.

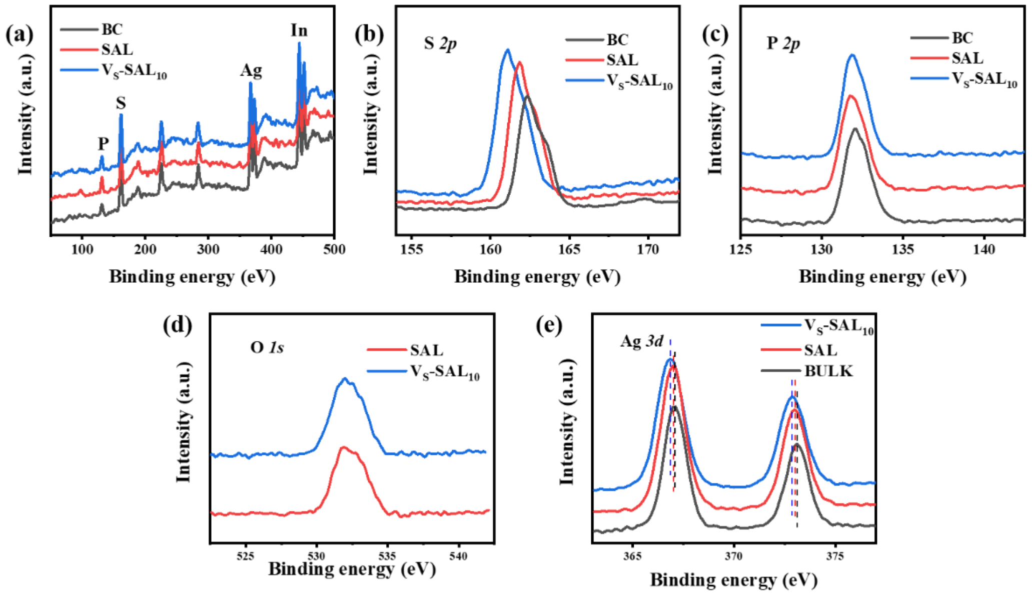


**Supplementary Figure 10.** XPS spectra of BC, SAL, and $V_S$-$SAL_{10}$. (a) Full XPS spectra of BC, SAL, and $V_S$-$SAL_{10}$. High-resolution (b) S *2p* and (c) P *2p* XPS spectra of BC, SAL, and $V_S$-$SAL_{10}$. (d) High-resolution O *1s* XPS spectra of SAL and $V_S$-$SAL_{10}$. (e) High-resolution Ag 3*d* XPS spectra of BC, SAL, and $V_S$-$SAL_{10}$.

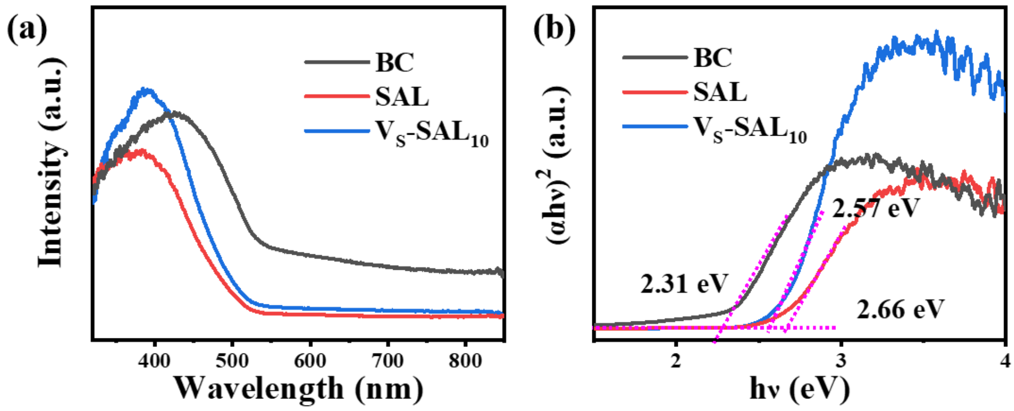


**Supplementary Figure 11.** (a) UV-visible absorption spectra and (b) the corresponding band edge (b) of BC, SAL, and $V_s$-$SAL_{10}$.

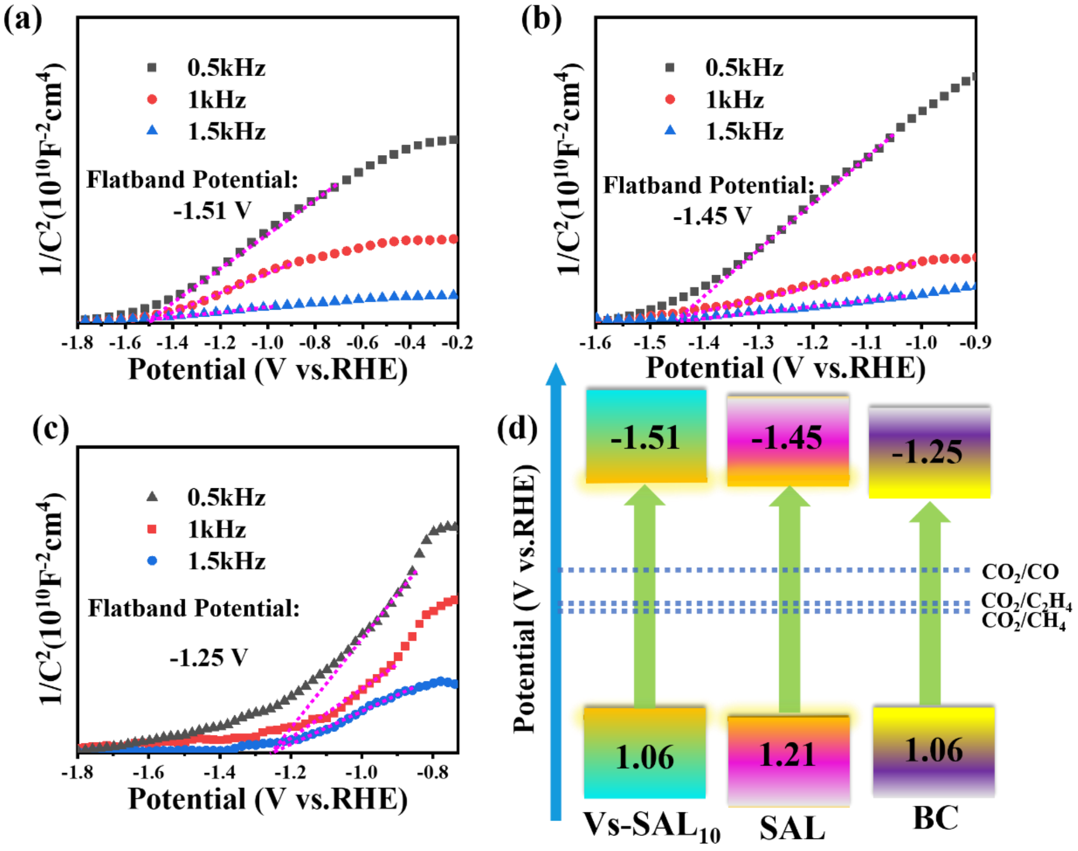


**Supplementary Figure 12.** Electronic band structure characterization. Mott-Schottky plots of (a) Vs-$SAL_{10}$, (b) SAL, and (c) BC. (d) Schematics illustrating the electronic band structures.

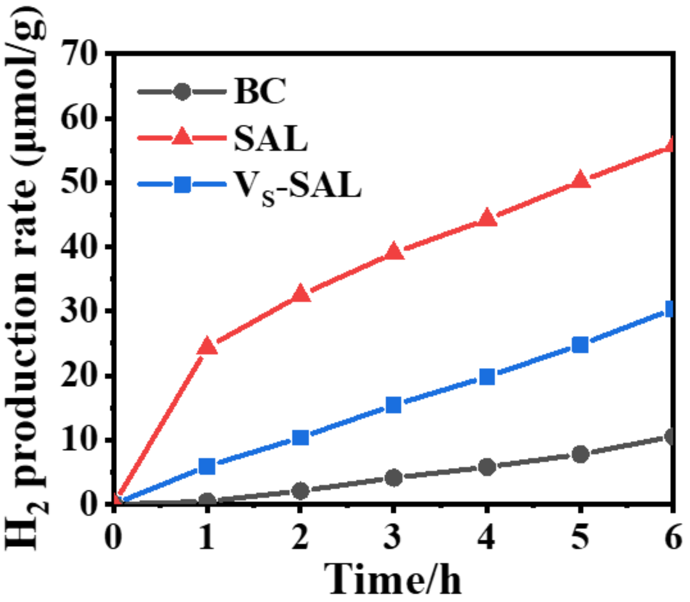


**Supplementary Figure 13.** Photocatalytic $H_2$ evolution amounts as a function of light irradiation times of BC, SAL, and $V_S$-$SAL_{10}$.

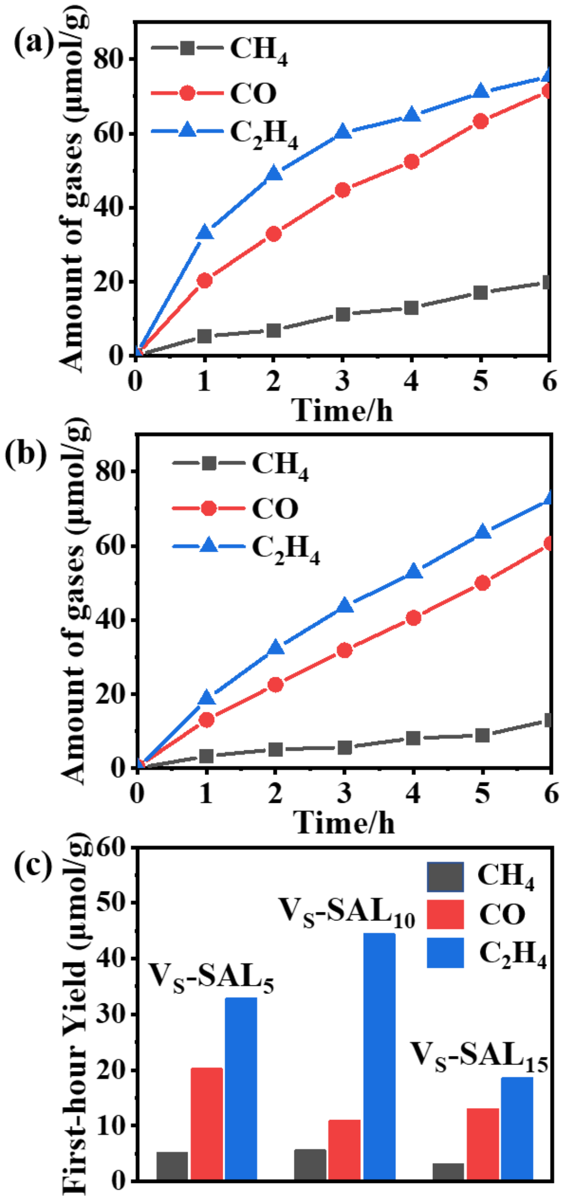


**Supplementary Figure 14.** Photocatalytic $CO_2$ reduction performances. Photocatalytic gasses evolution amounts as a function of light irradiation times of (a) $V_S$-$SAL_5$ and (b) $V_S$-$SAL_{15}$. (c) Comparison of photocatalytic activity of $V_S$-$SAL_5$, $V_S$-$SAL_{10}$, and $V_S$-$SAL_{15}$ for the first hour.

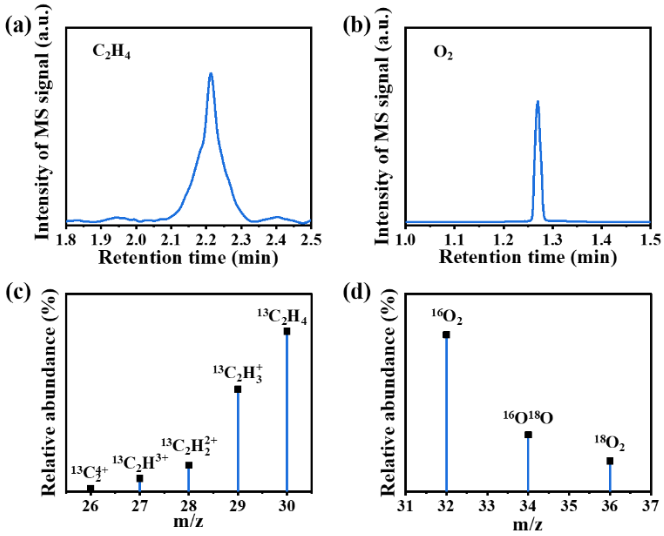


**Supplementary Figure 15.** Gas chromatogram traces (a, b) and mass spectra of $^{13}C_2H_4$ and $^{18}O_2$ (c, d) produced over $V_S$-$SAL_{10}$. $^{13}CO_2$ and $H_2{}^{18}O$ were used for $^{13}C_2H_4$ and $^{18}O_2$ detection, respectively.

Isotropic experiments using $^{13}CO_2$ are performed to investigate the carbon source of the products. As shown in Figure S12(a), the peak at m/z = 30 is the base peak of $^{13}C_2H_4$; meanwhile the peak at m/z = 29, 28, 27, 26 can be assigned to $^{13}C_2H_3{}^+$, $^{13}C_2H_2{}^{2+}$, $^{13}C_2H^{3+}$ and $^{13}C_2{}^{4+}$, respectively, which are the fragments of $^{13}C_2H_4$. This unambiguously confirms that the carbon source of $C_2H_4$ is the introduced $CO_2$.[1-5]

$O_2$ generation was traced using the isotope $H_2{}^{18}O$. $^{16}O_2$ (m/z = 32), $^{18}O^{16}O$ (m/z = 34), and $^{18}O_2$ (m/z = 36) were simultaneously detected from the reaction (Figure S12b). It indicates that the product molecular oxygen is derived from both initially added $H_2{}^{18}O$ and the $C^{16}O_2$ reduction. The *in situ* formed $H_2{}^{16}O$ from the $C^{16}O_2$ reduction, which is more easily oxidized than the added $H_2{}^{18}O$ in gas phase, may be mainly responsible for $^{16}O_2$.[1] The possible $O_2$ leakage from air can be not also excluded with our present measurement condition. The photocatalytic gas oxygen isotope exchange between $^{18}O_2$ and $^{16}O_2$, may leads to the formation of $^{18}O^{16}O$ ($^{18}O_2 + {}^{16}O_2 \rightarrow 2\ {}^{18}O^{16}O$).[6-8]

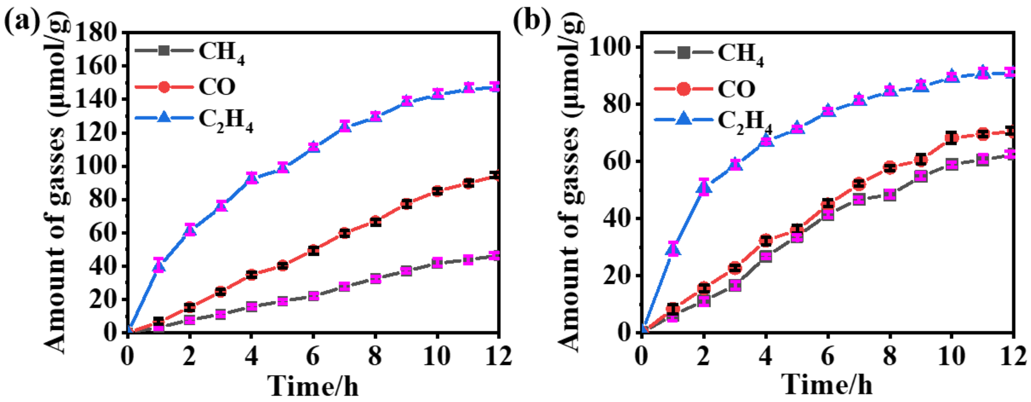


**Supplementary Figure 16.** The stability of Vs-SAL$_{10}$ during the photocatalytic reaction. Photocatalytic $CO_2$ activity of (a) fresh Vs-SAL$_{10}$ and (b) post washing treated-Vs-SAL$_{10}$ after 12-hour photocatalysis reaction. It should be mentioned that the deposited carbon can not be absolutely removed in the present case. More effective methods to completely re-expose activity sites for recycle utilization of the photocatalyst is still explored currently.

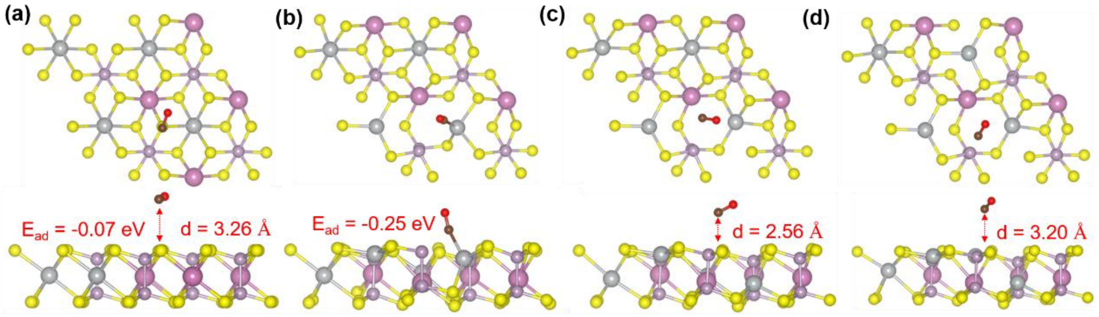


**Supplementary Figure 17.** Structure (top and side views) of CO molecule adsorbed on (a) defect-free $AgInP_2S_6$ surface and (b) exposed Ag sites (c) exposed In sites (d) exposed P sites, respectively. Sulfur, phosphorus, indium, silver, carbon, oxygen, and hydrogen atoms are yellow, purple, lilac, gray, black, red, and white, respectively. The adsorption energy $E_{ad}$ was calculated by a standard formula: $E_{ad}= E_{catalyst + CO}-E_{catalyst}-E_{CO}$, where $E_{catalyst}$ and $E_{catalyst + CO}$ are the energies of pristine or S-vacancy $AgInP_2S_6$ before and after adsorption of a single CO molecule and $E_{molecule}$ is the energy of CO molecule.

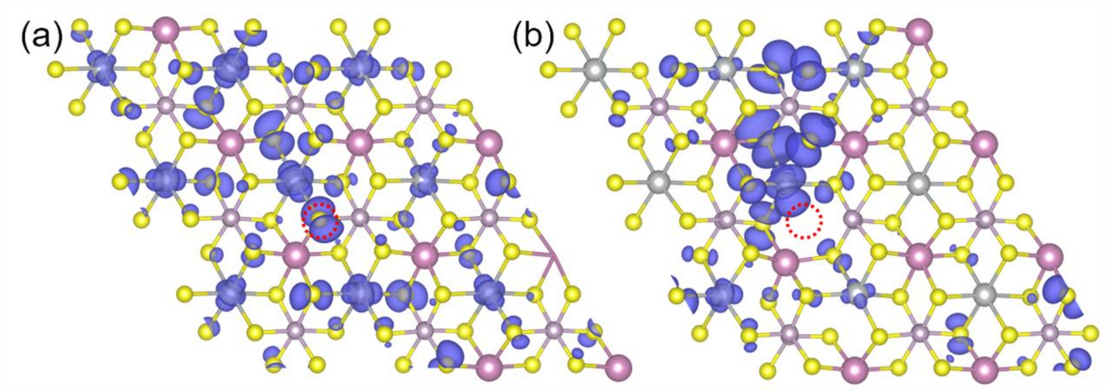


**Supplementary Figure 18.** Crystal structure with charge density contour plots of (a) the $V_S$-free and (b) the $V_S$-$AgInP_2S_6$ supercell. The blue area represents enrichment of the charge, showing charge enrichment on the Ag atoms near the S vacancies. The isosurface value is set at 0.001 e/$Bohr^3$.

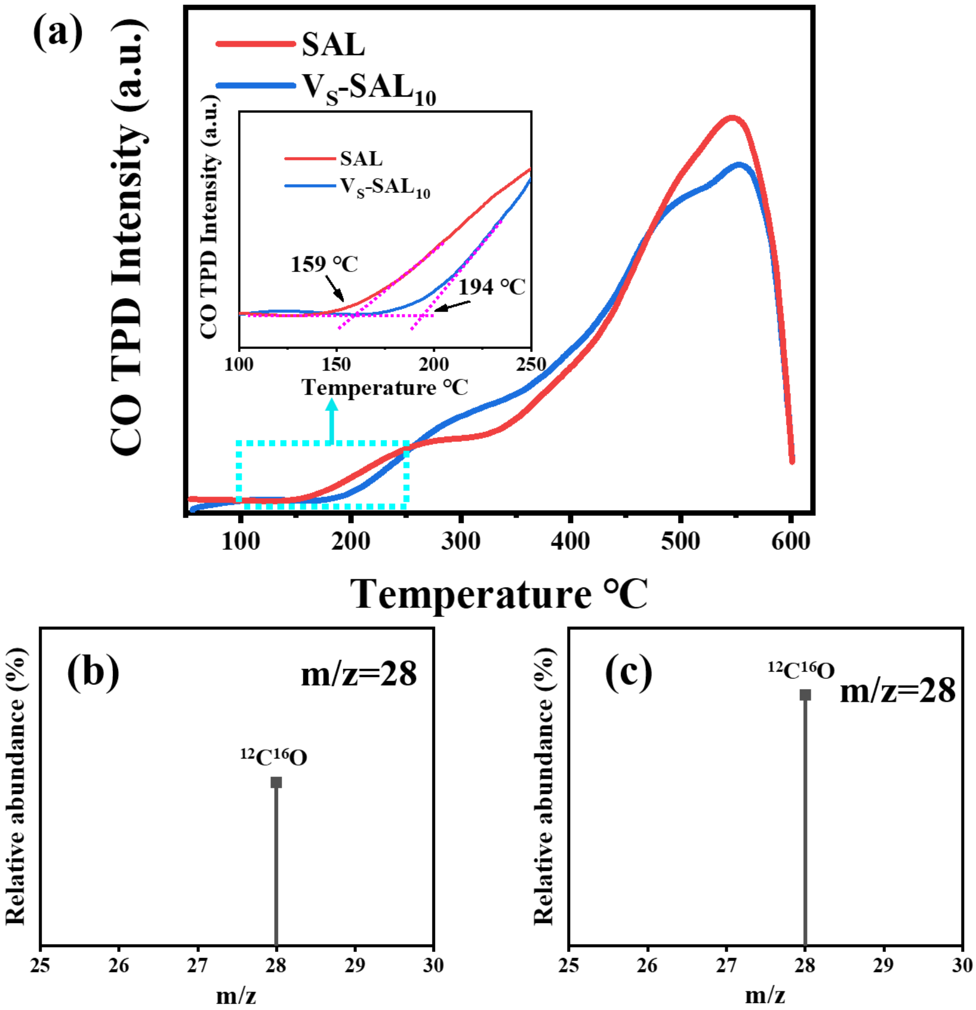


**Supplementary Figure 19.** Analysis of CO TPD of SAL and $V_S$-$SAL_{10}$. (a) CO TPD spectra of SAL and $V_S$-$SAL_{10}$. (b, c) Mass spectra of corresponding TPD peaks of CO desorbed from SAL and $V_S$-$SAL_{10}$, respectively.

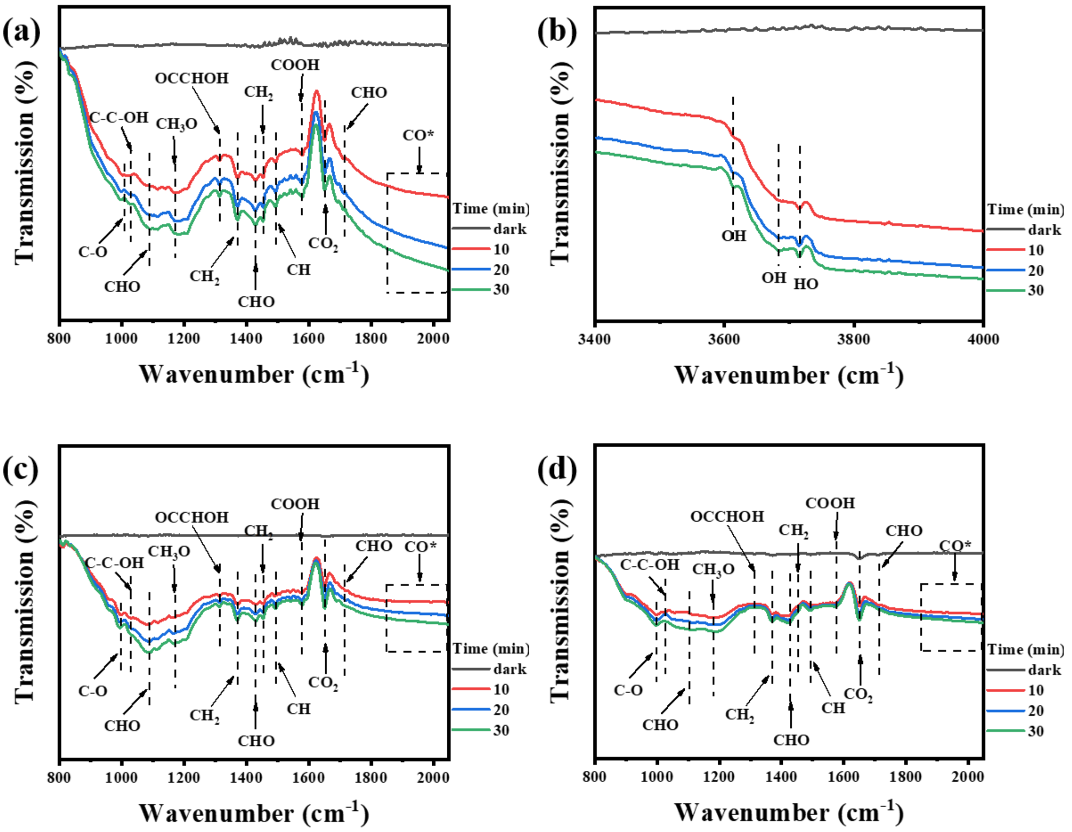


**Supplementary Figure 20.** *In situ* FTIR characterization. *In situ* FTIR spectra for the adsorption and activation of $CO_2$ on (a, b) $V_S$-$SAL_{10}$, (c) SAL and (d) BC, respectively.

*In situ* FTIR measurements were performed to trace the proposed reaction intermediates. Those FTIR peak intensities gradually increases with extension of the irradiation time for BC, SAL, and $V_S$-$SAL_{10}$. A characteristic peak at around 1580 $cm^{-1}$ can be ascribed to COOH* groups, the crucial intermediate for reducing $CO_2$ to $C_1$ and multi-carbon products.[9,10] The peaks at around 1085, 1425 and 1714 $cm^{-1}$ may be ascribed to CHO.[11-13] The peaks at around 1170 $cm^{-1}$ may be ascribed to $CH_3O$, the intermediate of $CH_4$.[14,10] These COOH, CHO and $CH_3O$ peaks are more prominently for both $V_S$-$SAL_{10}$ and SAL, compared with BC, further demonstrating that the atomically thin nano-geometry is greatly favorable for activation and catalytic

reduction process of the adsorbed $CO_2$. The characteristic peak between about 2050 and 2250 $cm^{-1}$ is assigned to CO*.[15,16] The relative weaker peak intensity for SAL compared with $V_s$-$SAL_{10}$ demonstrates that CO* could indeed easier desorb from SAL surface to form free CO molecules to be conducive to the product of CO.[17] Furthermore, the absorption peaks developed at around 1372, 1452 and 1492 $cm^{-1}$, which can be attributed to the different vibration of $CH_2$ and CH, may reflect the generation of $CH_4$ and $C_2H_4$.[18] The peak of the intermediate groups at around 1025 and 1312 $cm^{-1}$ detailedly ascribing to C-C-OH and OCCHOH, respectively, may reflect the generation of $C_2$ product with obviously stronger intensity for $V_S$-$SAL_{10}$ than SAL, authenticating the profit of S vacancy defects for the C–C dimerization products generation.[19-23] Meanwhile, the bands at 3613, 3683 and 3713$cm^{-1}$ were assigned to the OH may originate from the generation of CH-CHOH and CH-$CH_2$OH, and reactant of $H_2O$.[24]

$$CO_2 + H^+ + e^- + * \rightarrow *COOH \quad (1)$$

$$*COOH + e^- + H^+ \rightarrow *CO + H_2O \quad (2)$$

$$*CO + e^- + H^+ \rightarrow *CHO \text{ or } *CO \rightarrow CO\uparrow + * \quad (3)$$

$$*CHO + 5e^- + 5H^+ \rightarrow *CH_4\uparrow + H_2O \quad (4)$$

$$*CHO + e^- + H^+ \rightarrow *CHOH \quad (5)$$

$$*CHOH + *CO \rightarrow *CHOH\text{-}*CO \quad (6)$$

$$*CHOH\text{-}*CO + H^+ + e^- \rightarrow *CHOH\text{-}CHO + * \quad (7)$$

$$*CHOH\text{-}CHO + H^+ + e^- \rightarrow *CH\text{-}CHO + H_2O \quad (8)$$

$$*CH\text{-}CHO + H^+ + e^- \rightarrow *CH\text{-}CHOH \quad (9)$$

$$*CH\text{-}CHOH + H^+ + e^- \rightarrow *CH\text{-}CH_2OH \quad (10)$$

$$*CH\text{-}CH_2OH + H^+ + e^- \rightarrow *CH\text{-}CH_2 + H_2O \quad (11)$$

$$*CH\text{-}CH_2 + H^+ + e^- \rightarrow *CH_2\text{-}CH_2 \rightarrow CH_2\text{-}CH_2\uparrow \quad (12)$$

**Supplementary Figure 21.** The reaction process for reduction of $CO_2$ into CO, $CH_4$ and $C_2H_4$ over $V_S$-$SAL_{10}$ under light illumination.

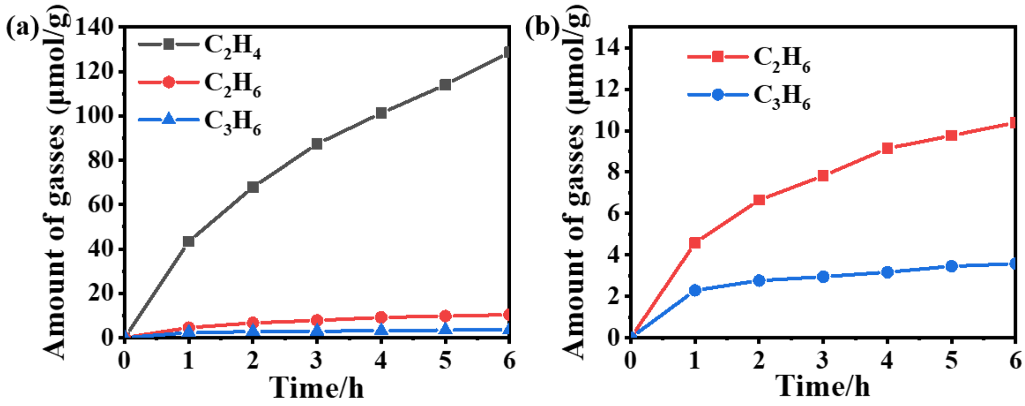


**Supplementary Figure 22.** Photocatalytic gasses evolution amounts as a function of light irradiation times of $V_S$-$SAL_{10}$ using CO as starting reactants.

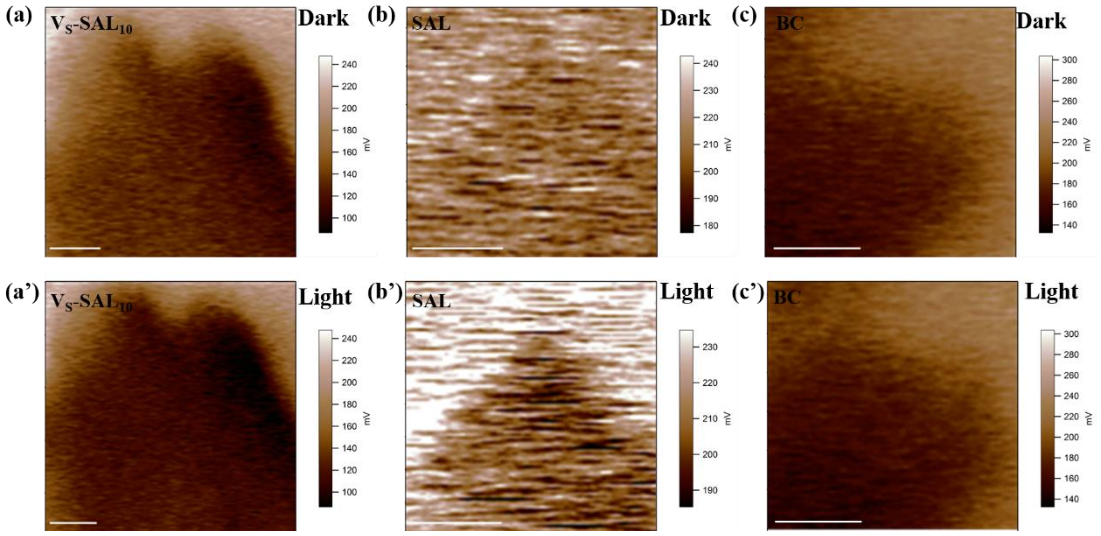


**Supplementary Figure 23.** SPV characterization. Surface potential images of $V_S$-$SAL_{10}$ (a) before and (a’) after light illumination, surface potential images of SAL (b) before and (b’) after light illumination, surface potential images of BC (c) before and (c’) after light illumination.

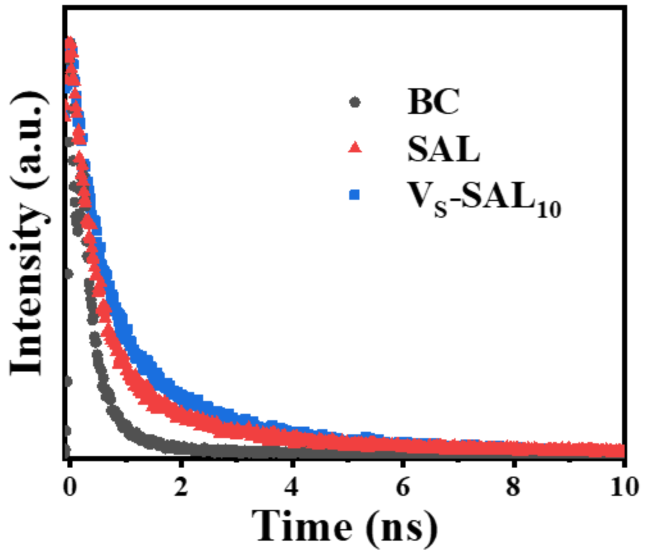


**Supplementary Figure 24.** Time-resolved PL decay spectra for BC, SAL and $V_S$-$SAL_{10}$.

**Supplementary Table 1**. The fitted results of the time-resolved PL.

| | $A_1$ | $t_1$ | $A_2$ | $t_2$ | $t_{average}$ |
|---|---|---|---|---|---|
| BC | 0.55992 | 0.39691 | 0.34462 | 0.39693 | 0.39692 |
| SAL | 0.7897 | 0.41421 | 0.21536 | 2.01228 | 1.3249 |
| $V_S$-$SAL_{10}$ | 0.68401 | 0.50206 | 0.31465 | 2.0364 | 1.50101 |

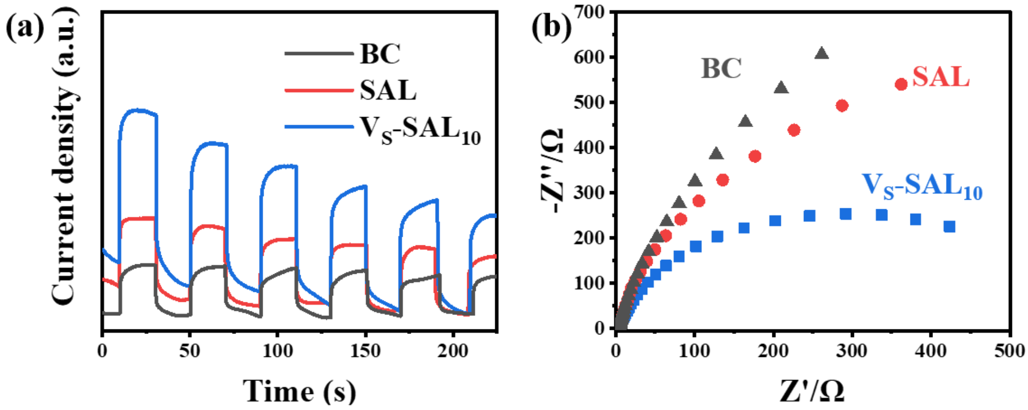


**Supplementary Figure 25.** Transient photocurrent responses (a) and EIS (b) of BC, SAL, and $V_S$-SAL$_{10}$.

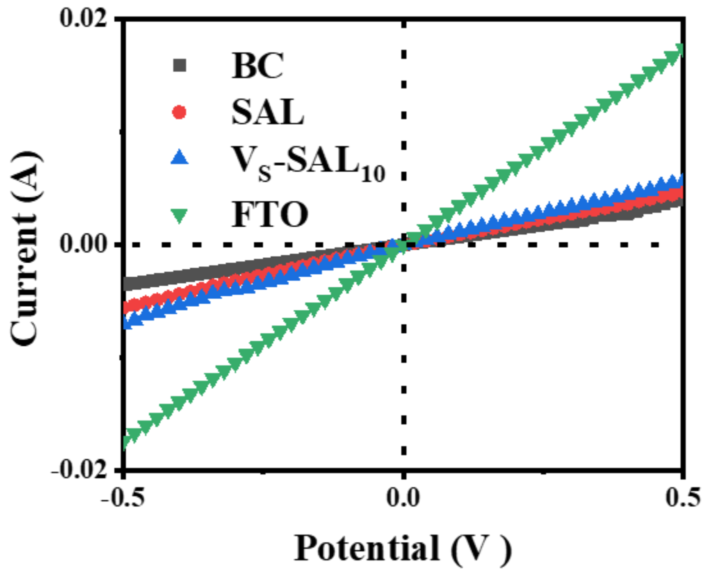


**Supplementary Figure 26.** Solid-state current-voltage curves of BC, SAL, and $V_S$-$SAL_{10}$.

## Supplementary References


(1) Jiang, W. et al. Pd-Modified ZnO–Au Enabling Alkoxy Intermediates Formation and Dehydrogenation for Photocatalytic Conversion of Methane to Ethylene. *J. Am. Chem. Soc.* **143,** 269-278 (2021).

(2) Xia, Y. et al. Improving Artificial Photosynthesis over Carbon Nitride by Gas–Liquid–Solid Interface Management for Full Light-Induced $CO_2$ Reduction to $C_1$ and $C_2$ Fuels and $O_2$. *ChemSusChem* **13**, 1730–1734 (2020).

(3) Li, N. et al. Plasma-Assisted Photocatalysis of $CH_4$ and $CO_2$ into Ethylene. *ACS Sustain. Chem. Eng.* **7**, 11455–11463 (2019).

(4) Matilainen, A., Gjessing, E. T., Lahtinen, T., Hed, L., Bhatnagar, A. & Sillanpää, M. An Overview of the Methods Used in the Characterisation of Natural Organic Matter (NOM) in Relation to Drinking Water Treatment. *Chemosphere* **83**, 1431–1442 (2011).

(5) Sela, P., Peukert, S., Herzler, J., Sakai, Y., Fikri, M. & Schulz, C. High-Temperature Gas-Phase Kinetics of the Thermal Decomposition of Tetramethoxysilane. *Proc. Combust. Inst.* **37**, 1133–1141 (2019).

(6) Wang, Y. et al. Visible-Light Driven Overall Conversion of $CO_2$ and $H_2O$ to $CH_4$ and $O_2$ on 3D-SiC@2D-$MoS_2$ Heterostructure. *J. Am. Chem. Soc.* **140**, 14595–14598 (2018).

(7) Tanaka, K. I. Intermediate of Oxygen Exchange Reaction over Illuminated Titanium Dioxide. *J. Phys. Chem.* **78**, 555–556 (1974).

(8) Sato, S. Hydrogen and Oxygen Isotope Exchange Reactions over Illuminated and Nonilluminated $TiO_2$. *J. Phys. Chem.* **91**, 2895–2897 (1987).

(9) Firet, N. J. & Smith, W. A. Probing the Reaction Mechanism of $CO_2$ Electroreduction over Ag Films via Operando Infrared Spectroscopy. *ACS Catal.*

**7**, 606–612 (2017).

(10) Xu, J. et al. Efficient Infrared‐Light‐Driven $CO_2$ Reduction Over Ultrathin Metallic Ni‐doped $CoS_2$ Nanosheets. *Angew. Chemie - Int. Ed.* **133**, 8787‐8791 (2021).

(11) Chen, X., Li, Y., Pan, X., Cortie, D., Huang, X. & Yi, Z. Photocatalytic Oxidation of Methane over Silver Decorated Zinc Oxide Nanocatalysts. *Nat. Commun.* **7**, 12273 (2016).

(12) Xiong, L. et al. Breaking the Linear Scaling Relationship by Compositional and Structural Crafting of Ternary Cu–Au/Ag Nanoframes for Electrocatalytic Ethylene Production. *Angew. Chemie - Int. Ed.* **60**, 2508–2518 (2021).

(13) Yee, A., Morrison, S. J. & Idriss, H. A Study of the Reactions of Ethanol on $CeO_2$ and Pd/$CeO_2$ by Steady State Reactions, Temperature Programmed Desorption, and in Situ FT-IR. *J. Catal.* **186**, 279–295 (1999).

(14) Li, X. et al. Selective Visible-Light-Driven Photocatalytic $CO_2$ Reduction to $CH_4$ Mediated by Atomically Thin $CuIn_5S_8$ Layers. *Nat. Energy*, **4**, 690–699 (2019).

(15) Wang, J. et al. Enabling Visible-Light-Driven Selective $CO_2$ Reduction by Doping Quantum Dots: Trapping Electrons and Suppressing $H_2$ Evolution. *Angew. Chemie - Int. Ed.* **130**, 16685–16689 (2018).

(16) Schnaidt, J., Heinen, M., Jusys, Z. & Behm, R. J. Oxidation of the Partly Oxidized Ethylene Glycol Oxidation Products Glycolaldehyde, Glyoxal, Glycolic Acid, Glyoxylic Acid, and Oxalic Acid on Pt Electrodes: A Combined ATR-FTIRS

and DEMS Spectroelectrochemical Study. *J. Phys. Chem. C* **117**, 12689–12701 (2013).

(17) Wang, J. et al. Enabling Visible-Light-Driven Selective $CO_2$ Reduction by Doping Quantum Dots: Trapping Electrons and Suppressing $H_2$ Evolution. *Angew. Chemie - Int. Ed.* **57,** 16447–16451 (2018).

(18) Zhang, R. et al. Photocatalytic Oxidative Dehydrogenation of Ethane Using $CO_2$ as a Soft Oxidant over Pd/$TiO_2$ Catalysts to $C_2H_4$ and Syngas. *ACS Catal.* **8**, 9280–9286 (2018).

(19) Karimi, S., Feizy, J., Mehrjo, F. & Farrokhnia, M. Detection and Quantification of Food Colorant Adulteration in Saffron Sample Using Chemometric Analysis of FT-IR Spectra. *RSC Adv.* **6**, 23085–23093 (2016).

(20) Fan, M., Dai, D. & Huang, B. Fourier Transform Infrared Spectroscopy for Natural Fibres. Fourier Transform - Mater. *Anal.* 2012. https://doi.org/10.5772/35482.

(21) Pérez-Gallent, E., Figueiredo, M. C., Calle-Vallejo, F. & Koper, M. T. M. Spectroscopic Observation of a Hydrogenated CO Dimer Intermediate During CO Reduction on Cu(100) Electrodes. *Angew. Chemie - Int. Ed.* **56**, 3621–3624 (2017).

(22) Yang, Y. P., Zhang, Y., Lang, Y. X. & Yu, M. H. Structural ATR-IR Analysis of Cellulose Fibers Prepared from a NaOH Complex Aqueous Solution. *IOP Conf. Ser. Mater. Sci. Eng.* **213**, 012039 (2017).

(23) Heredia-Guerrero, J. A. et al. Infrared and Raman Spectroscopic Features of Plant Cuticles: A Review. *Front. Plant Sci.* **5**, 1–14 (2014).

(24) Kähler, K., Holz, M. C., Rohe, M., Strunk, J. & Muhler, M. Probing the Reactivity of ZnO and Au/ZnO Nanoparticles by Methanol Adsorption: A TPD and DRIFTS Study. *ChemPhysChem* **11**, 2521–2529 (2010).
